# The shifting burden of tracheal, bronchus and lung cancer in ageing Asia: structural drivers and projections to 2050

**DOI:** 10.1080/07853890.2026.2722478

**Published:** 2026-09-11

**Authors:** Lei Zhu, Linping Fan, Jiangna Wen, Suqin Peng, Xianqiang Luo, Xuan Zhang

**Affiliations:** ^a^ Center for Molecular Diagnostics and Precision Medicine, The First Affiliated Hospital of Nanchang University, Nanchang, Jiangxi, China; ^b^ Jiangxi Human Resources Co., Ltd, Nanchang, Jiangxi, China

**Keywords:** Ageing population, disease burden, Asia, demographic decomposition, projections

## Abstract

**Background:**

Tracheal, bronchus, and lung (TBL) cancer remains a major cause of cancer morbidity and mortality. We quantified historical trends, structural drivers, inequality, and future burden of TBL cancer among Asian adults aged ≥60 years.

**Methods:**

Using Global Burden of Disease 2023 data, we assessed incidence, prevalence, mortality, and disability-adjusted life years (DALYs) from 1990 to 2023. Age-standardised rates (ASRs), estimated annual percentage changes, sex- and age-specific patterns, socioeconomic inequality, and demographic decomposition were evaluated. Bayesian age–period–cohort modelling projected burden to 2050.

**Results:**

From 1990 to 2023, incident cases increased from 334,563 to 1,102,794 and DALYs from 7.3 million to 19.4 million. ASR point estimates changed modestly, with Asia-wide EAPCs not statistically distinguishable from zero. Burden increased sharply with age and remained consistently higher in males. Socioeconomic inequality attenuated, particularly for mortality and DALYs. Population growth accounted for much of the absolute increase, while negative age-specific-rate components partly offset demographic effects in several Central Asian countries. From 2024 to 2050, ASIR and ASDR were projected to decline by 44.2% and 45.6%, respectively, with only modest reductions in absolute numbers.

**Conclusions:**

TBL cancer burden in older Asian adults is increasingly shaped by demographic expansion and heterogeneous epidemiological change. Despite projected declines in ASRs, population ageing will sustain substantial burden, underscoring the need for targeted prevention and age-responsive health-system planning.

## Introduction

Tracheal, bronchus and lung (TBL) cancer remains a leading cause of cancer-related morbidity and mortality worldwide, accounting for an estimated 2.2 million new cases and 1.8 million deaths annually [1,2]. In Asia, home to more than half of the global population, TBL cancer is increasingly shaped by population ageing, heterogeneous exposures and unequal health-system capacity [3,4].

Population ageing has become a defining demographic trend across the region. As life expectancy rises and fertility declines, the population aged 60 years and older is expanding rapidly, among whom TBL incidence, mortality and overall burden increase steeply [5]. More than 40% of TBL cancer cases now occur in adults aged 70 years and older, and this proportion is likely to rise further as the number of older adults in Asia continues to grow [5,6]. Smoking, air pollution, occupational risks and access to diagnosis and treatment vary widely across Asia, generating divergent burden patterns and persistent inequalities [7,8].

Despite previous Global Burden of Disease (GBD) analyses describing TBL cancer burden at global and regional levels [2,4], important gaps remain for older adults in Asia. Most studies have focused on all-age populations, leaving burden patterns in older adults insufficiently characterized [2,4]. The demographic and epidemiological components of changing burden remain poorly quantified. Cross-country inequality and future burden in ageing Asian populations also remain insufficiently characterized [6,7,9]. Using GBD 2023 data, we assessed TBL cancer incidence, prevalence, mortality and DALYs among adults aged 60 years and older in Asia from 1990 to 2023. We further examined sex-, age- and country-specific patterns, socio-economic inequality, decomposition components, and Bayesian age–period–cohort (BAPC) projections to 2050.

## Methods

### Study design and data sources

We did a comprehensive analysis of the burden of TBL cancer among older adults in Asia using data from the GBD 2023 study. GBD 2023 provides comparable estimates of incidence, prevalence, mortality and DALYs for 375 diseases and injuries across 204 countries and territories from 1990 to 2023. Detailed descriptions of the GBD analytical framework and estimation procedures have been reported previously [10]. Data were extracted from the Global Health Data Exchange (GHDx) results tool on 20 December, 2025; this access date defined the analytic version of the GBD 2023 estimates used in all analyses. Analyses were restricted to individuals aged 60 years and older in accordance with the United Nations definition of older adults [11].

### Case definition and measures

TBL cancer was defined according to the International Classification of Diseases, 10th revision (ICD-10), codes C33–C34. Four measures were analysed: incident cases, prevalent cases, deaths and DALYs. Age-standardized rates (ASRs) per 100 000 population were calculated using the direct method with the GBD world standard population [12]. For each measure, we use the terms age-standardized incidence rate (ASIR), age-standardized prevalence rate (ASPR), age-standardized death rate (ASDR) and the corresponding age-standardized DALYs rate.

Temporal trends in ASRs were assessed using the estimated annual percentage change (EAPC), derived from a log-linear regression model:

ln⁡(ASR)=α+β×year+ε

EAPC was calculated as 100×(exp⁡(β)−1), with 95% confidence intervals (CIs) derived from the standard error of β.

GBD estimates were reported with 95% uncertainty intervals (UIs), defined as the 2.5th and 97.5th percentiles of 1,000 posterior draws. EAPCs were summarized with 95% CIs.

### Geographical and demographic stratification

All Asian countries and territories defined by the GBD regional classification were included. Analyses were stratified by sex (both sexes, male and female) and by five-year age groups from 60–64 years to 95 years and older.

Socio-demographic Index (SDI), a composite indicator based on lag-distributed income per capita, mean educational attainment among individuals aged 15 years and older, and total fertility rate under age 25 years, was used to assess cross-country inequality [13]. Countries were ranked by SDI and grouped into quintiles.

### Statistical analysis

#### Decomposition analysis

To quantify the drivers of change in TBL cancer burden between 1990 and 2023, we applied a decomposition analysis based on the Das Gupta method [14]. The total change in the number of events was partitioned into three components:

$$\Delta N=N_{2023}-N_{1990}=\Delta N_{\text{population}}+\Delta N_{\text{ageing}}+\Delta N_{\text{epidemiology}}$$

where population growth reflects increases in total population size, population ageing reflects shifts in age structure towards older ages and epidemiological change represents changes in age-specific rates after accounting for demographic effects. Positive values indicate contributions to increasing burden, whereas negative values indicate reductions. This decomposition should be interpreted as a statistical accounting framework rather than a causal analysis. The estimated components represent the share of observed change in modelled event counts that was statistically attributed to changes in population size, age structure and age-specific rates under the decomposition algorithm; they do not establish causal effects of demographic or epidemiological factors.

#### Cross-country inequality analysis

Absolute and relative cross-country inequalities across the SDI gradient were assessed using the slope index of inequality (SII) and concentration index (CIX), respectively [15]. SII was estimated from a population-weighted linear regression of ASRs on the relative SDI rank, scaled from 0 to 1. Intuitively, SII represents the absolute difference in the age-standardized burden between countries at the lowest and highest ends of the SDI distribution; a positive SII indicates a higher burden in higher-SDI countries. CIX was derived from the concentration curve, which ranks countries by SDI and compares the cumulative share of population with the cumulative share of disease burden. CIX captures relative inequality: positive values indicate that the burden is disproportionately concentrated in higher-SDI countries, negative values indicate concentration in lower-SDI countries and values close to zero suggest a more even distribution across the SDI gradient. Ninety-five per cent CIs were estimated using bootstrap resampling with 1,000 replicates.

### Bayesian age–period–cohort projections

Future burden of TBL cancer among older adults was projected to 2050 using a BAPC model implemented with integrated nested Laplace approximation (INLA) [16]. Models were fitted separately by sex and measure using age-specific counts from 1990 to 2023 and corresponding population denominators; 2024–2050 was treated as the projection period.

Age-specific counts were modelled with a Poisson likelihood and the population at risk as the exposure. The latent log rate included age, period, cohort and overdispersion components. Age and cohort effects were specified as second-order random walks, period effects as first-order random walks and overdispersion as an iid random effect. Precision parameters for age, period and cohort effects used log-Gamma (1, 0.00005) priors, and overdispersion precision used a log-Gamma (1, 0.005) prior. These structured priors imposed temporal smoothness and reduced overfitting to short-term fluctuations. Because age, period and cohort are linearly dependent, the APC components were interpreted as structured latent terms for projection and temporal smoothing rather than as separately identifiable causal effects; identifiability was handled through the latent Gaussian model structure and internal constraints applied to the random-walk components.

Posterior uncertainty was propagated through the INLA posterior predictive distributions. Projected event numbers were obtained by applying projected age-specific rates to population forecasts from the UN World Population Prospects 2022 [17], and 95% prediction intervals were derived from posterior samples. Model adequacy and robustness were assessed using retrospective prediction, posterior predictive checks, prediction-interval review and prior-sensitivity analyses comparing the baseline prior with more flexible and smoother alternative prior specifications (Figure S1); full model specification and diagnostic results are provided in the Supplementary Methods.

### Software

All analyses were done in R version 4.3.2. Data management used dplyr and tidyr, visualization used ggplot2, Bayesian modelling used INLA and inequality analyses used ineq and dineq.

## Results

### Overall burden

From 1990 to 2023, the absolute burden of TBL cancer among older adults in Asia increased substantially (Table 1). Incident cases rose from 334,563 in 1990 to 1,102,794 in 2023, and deaths increased from 335,265 to 997,879. Prevalent cases increased from 354,595 to 1,514,849, alongside a rise in DALYs from 7,309,222 to 19,355,528. Over the same period, age-standardized point estimates changed more modestly. The ASIR was 139.68 per 100,000 in 1990 and 161.31 per 100,000 in 2023, whereas the age-standardized DALY rate was 3051.66 and 2831.12 per 100,000, respectively. However, the corresponding aggregate Asia-wide EAPCs were not statistically distinguishable from zero, and these rate changes should therefore be interpreted as descriptive point-estimate differences rather than confirmed directional trends. Across all indicators, males consistently experienced a substantially higher burden than females, with a male ASIR of 234.19 vs. 95.31 per 100,000 and a male age-standardized DALY rate of 4220.53 vs. 1573.16 per 100,000 in 2023.

**Table 1.** The burden of tracheal, bronchus and lung cancer in 1990 and 2023, and EAPC, by sex.

	1990		2023		EAPC (95%CI)
	Number (95%UI)	ASRs per 100 000 (95%UI)	Number (95%UI)	ASRs per 100 000 (95%UI)	
**Incidence**					
Both	334,563 (281,246, 427,217)	139.68 (117.42, 178.37)	1,102,794 (897,743, 1,301,995)	161.31 (131.31, 190.44)	1.94 (−8.12, 13.10)
Female	97,405 (77,920, 127,905)	78.07 (62.46, 102.52)	341,986 (264,567, 4,291,94)	95.31 (73.74, 119.62)	1.93 (−7.37, 12.17)
Male	237,158 (190,132, 326,030)	206.66 (165.68, 284.11)	760,808 (598,721, 936,117)	234.19 (184.30, 288.16)	1.95 (−7.25, 12.07)
**Prevalence**					
Both	354,595 (294,111, 452,068)	148.05 (122.79, 188.74)	1,514,849 (1,226,778, 1,873,967)	221.58 (179.44, 274.10)	2.88 (−7.27, 14.14)
Female	102,683 (81,751, 131,745)	82.30 (65.53, 105.60)	477,088 (357,947, 615,690)	132.97 (99.76, 171.59)	2.93 (−6.47, 13.27)
Male	251,913 (202,255, 350,269)	219.52 (176.25, 305.23)	1,037,762 (800,848, 1,315,161)	319.45 (246.52, 404.84)	2.87 (−6.42, 13.08)
**Death**					
Both	335,265 (281,910, 430,636)	139.98 (117.70, 179.79)	997,879 (817,699, 1,176,195)	145.96 (119.60, 172.04)	1.59 (−8.44, 12.70)
Female	98,500 (79,015, 129,982)	78.95 (63.33, 104.18)	309,420 (240,507, 384,714)	86.24 (67.03, 107.22)	1.55 (−7.72, 11.75)
Male	236,765 (190,474, 328,617)	206.32 (165.98, 286.36)	688,458 (535,832, 844,208)	211.92 (164.94, 259.87)	1.61 (−7.56, 11.70)
**DALYs**					
Both	7,309,222 (6,144,009, 9,384,463)	3,051.66 (2,565.17, 3,918.09)	19,355,528 (15,762,781, 23,111,242)	2,831.12 (2,305.61, 3,380.47)	1.22 (−8.77, 12.30)
Female	2,062,999 (1,642,850, 2,727,427)	1653.56 (1,316.80, 2,186.12)	5,644,588 (4,439,943, 7,122,232)	1,573.16 (1,237.42, 1,984.98)	1.11 (−8.12, 11.26)
Male	5,246,222 (4,218,086, 7,331,698)	4,571.66 (3,675.72, 6,388.98)	13,710,940 (10,506,289, 17,020,731)	4220.53 (3,234.07, 5,239.36)	1.27 (−7.87, 11.32)

Abbreviations: UI, uncertainty interval; CI, confidence interval; ASR, Age‑standardised rate; EAPC, estimated annual percentage change; DALYs, Disability-Adjusted Life Years.

### Country-level burden

**Figure 1.**
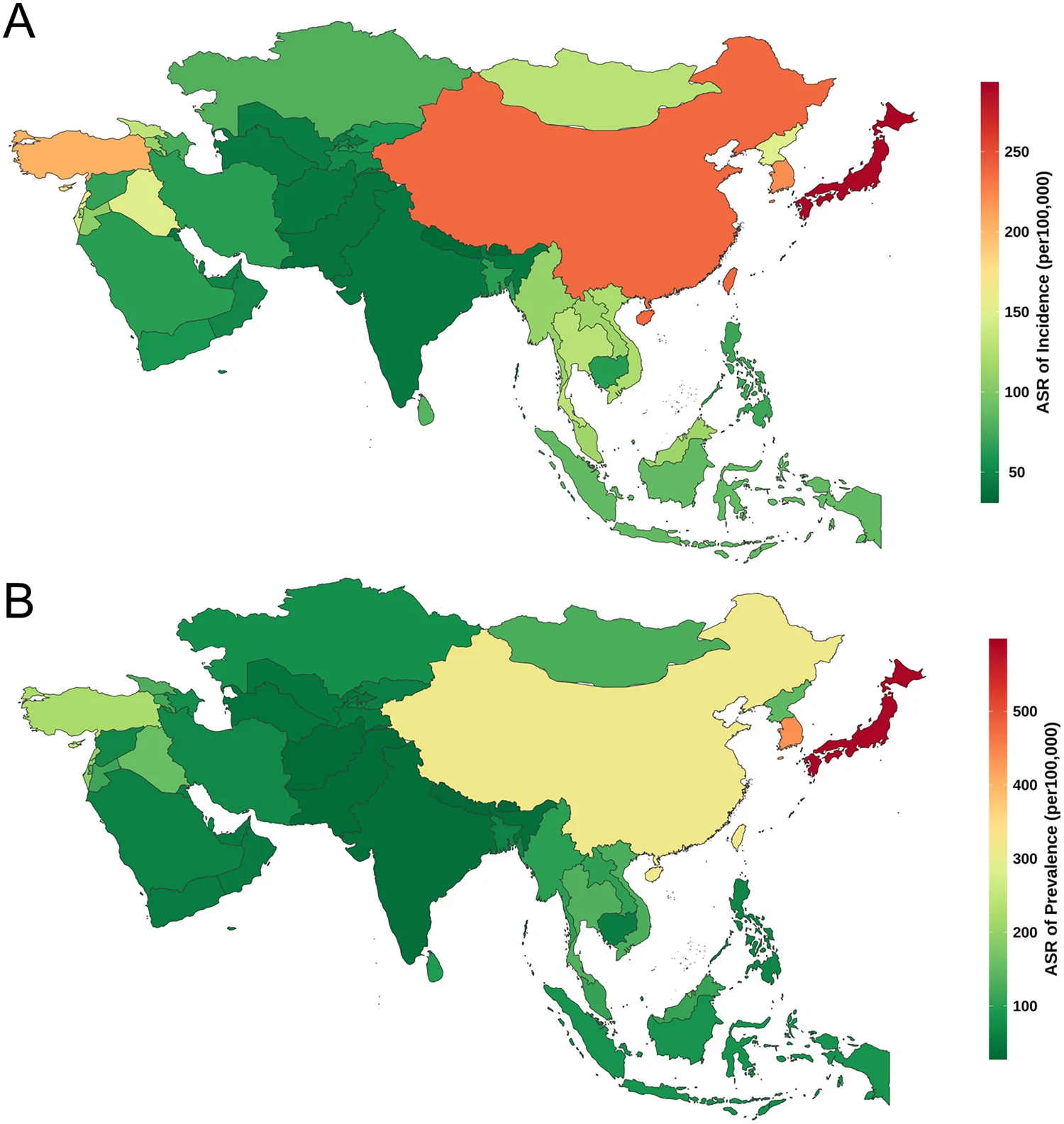
Age‑standardized rates of tracheal, bronchus and lung cancer among older adults in Asia, 2023. (A) Incidence. (B) Prevalence. Abbreviations: ASR, Age‑standardized rate.

Marked heterogeneity in the burden of TBL cancer among older adults was observed across Asia in 2023 (Figure 1; Figure S2; Tables S1–S4). Country-level rankings should be interpreted cautiously, because low estimated rates in settings with limited diagnostic capacity, cancer registration or cause-of-death certification may partly reflect under-ascertainment rather than genuinely low underlying risk. For incidence, the highest ASIRs were observed in Japan (290.14 per 100,000), China (236.88) and the Republic of Korea (220.52), whereas the lowest were recorded in Bhutan (31.69), Nepal (33.07) and Pakistan (38.70) (Figure 1A; Table S1). Prevalence showed a similar geographic pattern (Figure 1B; Table S2). In 2023, ASPR was the highest in Japan (591.54 per 100,000), the Republic of Korea (437.62) and China (318.96), and the lowest in Nepal (30.51), Afghanistan (33.54) and Bhutan (28.80).

Mortality and DALY patterns were similarly aligned (Figure S2; Tables S3–S4). The highest ASDRs in 2023 were in Japan (220.12 per 100,000) and China (212.54), with comparably high rates in Türkiye (202.64) and the Republic of Korea (167.38); by contrast, the lowest ASDRs were observed in Nepal (34.92), Kuwait (34.98), India (43.23) and Afghanistan (46.72) (Table S3). Across the study period, several Central Asian countries – most notably Kazakhstan, Kyrgyzstan and Uzbekistan – showed consistently declining ASRs across incidence, prevalence, deaths and DALYs, contrasting with increases in several West Asian and East Asian settings (Table S4).

### Sex- and age-specific trends

**Figure 2.**
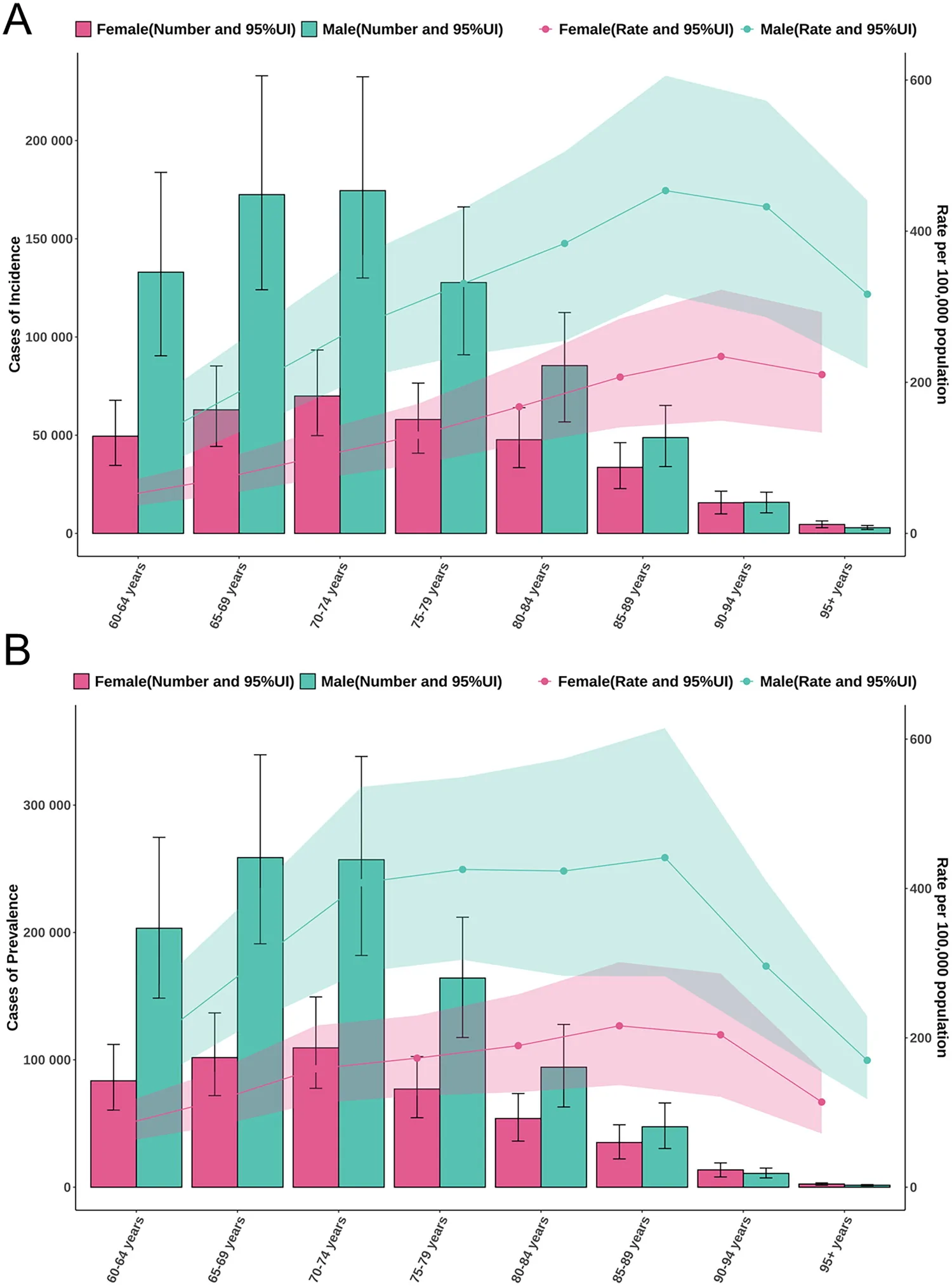
Age‑specific number and rates of tracheal, bronchus and lung cancer among older adults in Asia, 2023, by sex and age group. (A) Incidence. (B) Prevalence.

**Figure 3.**
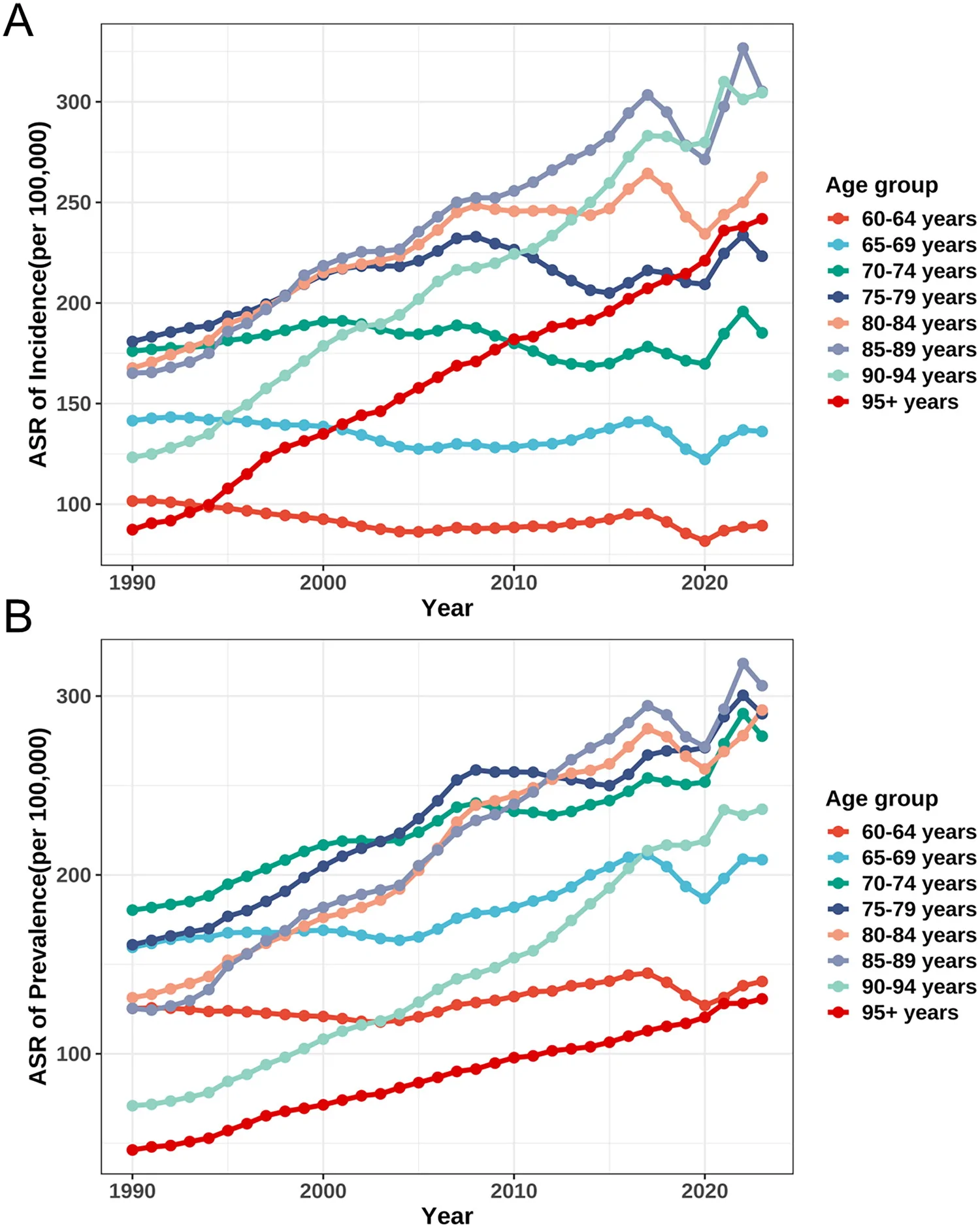
Age‑specific trends of tracheal, bronchus and lung cancer among older adults in Asia, 1990–2023. (A) Incidence. (B) Prevalence. Abbreviations: ASR, Age‑standardized rate.

Across 1990–2023, TBL burden increased steeply with age and remained consistently higher in males than females (Figure 2–3; Figure S3-S4; Tables S5–S8). For incidence, age-specific rates rose monotonically with age in both sexes, peaking in males aged 85–89 years (453.70 per 100,000) and in females aged 90–94 years (234.30 per 100,000) in 2023 (Table S5). Increases were concentrated in the oldest age groups; for example, among those aged 80–84 years, incidence rose from 258.27 to 383.81 per 100,000 in males and from 104.50 to 167.64 per 100,000 in females. Consistent with this shift toward the oldest-old, EAPCs increased with age, reaching 5.86/5.84 (males/females) at 90–94 years and 6.33/6.88 at ≥95 years.

A similar age escalation was observed for prevalence, with peak rates in 2023 at 85–89 years (305.83 per 100,000 overall; 441.39 in males vs. 216.15 in females), and a marked age gradient in EAPC from 1.85 at 60–64 years to 7.03 at 90–94 years and 6.75 at ≥95 years (Table S6). Mortality and DALYs mirrored these patterns: deaths and rates increased sharply with age (e.g. ASDR 385.78 per 100,000 at ≥95 years), and the fastest increases were consistently seen in the oldest-old (EAPCs at ≥95 years: 6.10 in males and 6.42 in females for mortality; DALY-rate EAPCs: 5.09 at 90–94 years and 6.23 at ≥95 years) (Tables S7–S8). Overall, these findings indicate an increasing concentration of TBL burden in the oldest-old, alongside persistent male predominance across age strata.

### Cross-country inequality in TBL burden

**Figure 4.**
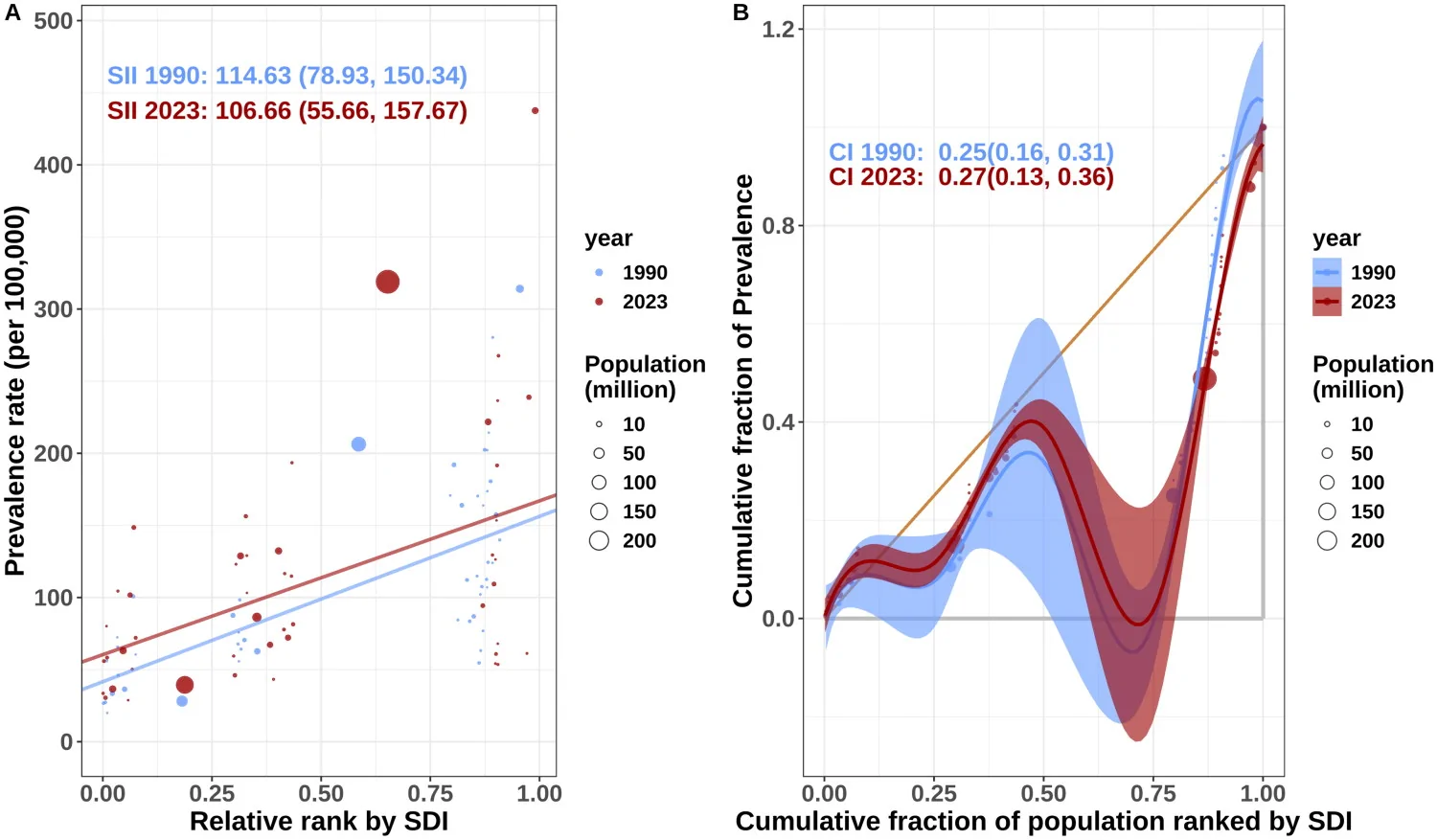
Cross-country inequality in tracheal, bronchus and lung cancer incidence among older adults in Asia, 1990 and 2023. Abbreviations: SDI, Socio-demographic Index.

Overall, socio-economic-related inequality in TBL burden across the SDI gradient attenuated between 1990 and 2023, most prominently for mortality and DALYs, whereas prevalence showed comparatively stable disparities (Figure 4; Figure S5–S7). This attenuation indicates reduced concentration of burden in higher-SDI countries, but should not be interpreted as uniform improvement across settings. For incidence, SII decreased from 112.27 per 100,000 (95% CI 76.25–148.28) in 1990 to 68.43 per 100,000 (26.27–110.60) in 2023; CIX also declined from 0.22 (0.14–0.28) to 0.15 (0.07–0.23) (Figure 4). For prevalence, SII showed only a modest reduction from 114.63 per 100,000 (78.93–150.34) to 106.66 per 100,000 (55.66–157.67), with overlapping CIs suggesting broadly stable absolute inequality, while CIX remained largely unchanged (0.25 [0.16–0.31] vs. 0.27 [0.13–0.36]) (Figure S5).

For mortality, SII declined from 111.54 per 100,000 (74.99–148.10) to 53.48 per 100,000 (12.44–94.53), and CIX decreased from 0.21 (0.14–0.27) to 0.11 (0.03–0.19) (Figure S6). For DALYs, SII decreased from 2416.71 per 100,000 (1642.31–3191.11) to 816.11 per 100,000 (81.68–1550.55), and CIX from 0.20 (0.13–0.26) to 0.08 (0.01–0.15) (Figure S7). These patterns may reflect both stabilization in some higher-SDI settings and accumulating burden in lower- or middle-SDI countries, suggesting inequality attenuation with possible epidemiological catch-up.

### Decomposition analysis

For incidence, the population-size component accounted for the largest statistically attributed share in most countries. In Turkmenistan (345.80%), the United Arab Emirates (340.03%) and Tajikistan (291.85%), this component exceeded the net increase, indicating offsetting by other components (Tables S9). The age-specific-rate component was positive in Afghanistan (40.19%), Bangladesh (37.81%) and Lebanon (46.27%), but negative in Kazakhstan (−263.96%) and Kyrgyzstan (−724.83%).

Prevalence showed a similar pattern, with the population-size component accounting for most net increases (Tables S10). Negative age-specific-rate components in Central Asia, particularly Kazakhstan, Kyrgyzstan and Uzbekistan, partly offset demographic components.

For mortality, demographic components accounted for much of the observed change, with the age-structure component more prominent than for incidence (Tables S11). In Japan, this component accounted for 31.00% of mortality change versus 21.93% for incidence. Negative age-specific-rate components in Central Asia similarly attenuated mortality increases. DALYs showed the largest absolute increases in populous countries (Tables S12). China had the greatest net increases, including +490 900 incident cases, +736 288 prevalent cases, +414 281 deaths and +7 462 583 DALYs. Overall, these findings reflect statistical attribution rather than causal effects.

### Future trends predicted by BAPC

**Figure 5.**
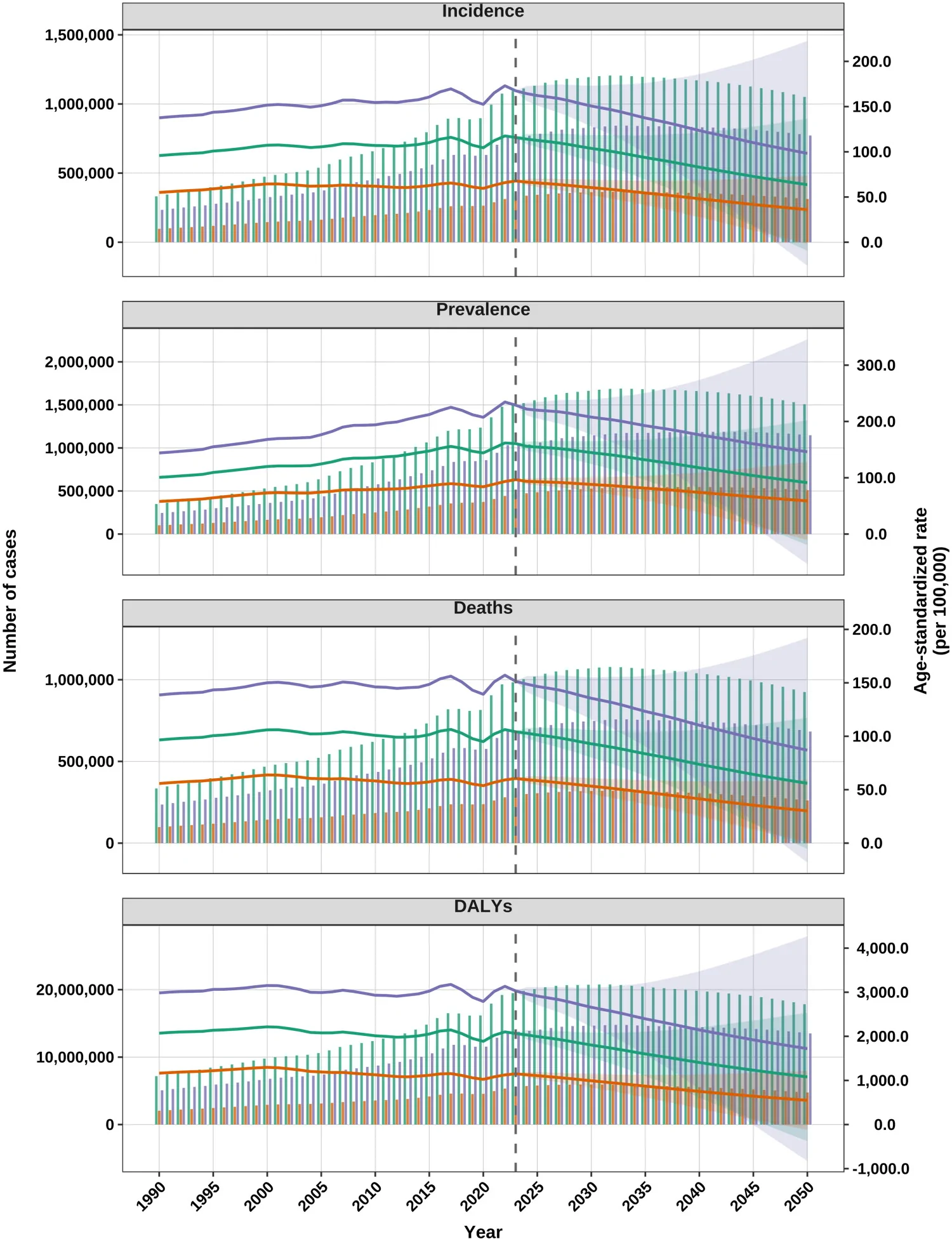
Projected age-standardised rates and numbers of tracheal, bronchus and lung cancer among older adults in Asia, 1990–2050. Abbreviations: DALYs, Disability-Adjusted Life Years.

Observed GBD estimates were available for 1990–2023, and BAPC projections were generated for 2024–2050 (Figure 5). Values from 2024 onward therefore represent model-derived projections rather than direct GBD observations.

Within the projection period, age-standardized rates were projected to decline across all four measures. The ASIR decreased from 114.12 per 100,000 in 2024 to 63.66 per 100,000 in 2050, corresponding to a 44.2% reduction. Similar reductions were projected for prevalence, mortality and DALYs: ASPR declined from 155.97 to 91.35 per 100,000 (−41.4%), ASDR from 102.92 to 55.98 per 100,000 (−45.6%) and the age-standardized DALY rate from 2033.42 to 1080.59 per 100,000 (−46.9%). Relative to the observed 2023 GBD baseline, the projected 2050 age-standardized rates were lower by 60.5% for incidence, 58.8% for prevalence, 61.6% for mortality and 61.8% for DALYs.

Projected absolute numbers declined more modestly. From 2024 to 2050, incident cases decreased from 1,112,491 to 1,051,109 (−5.5%), prevalent cases from 1,520,556 to 1,508,308 (−0.8%), deaths from 1,003,326 to 924,316 (−7.9%) and DALYs from 19,823,403 to 17,841,978 (−10.0%). These patterns suggest that demographic momentum may partly counterbalance projected reductions in age-specific risk.

## Discussion

Our study provides a comprehensive assessment of the burden, distribution and future trajectory of TBL cancer among older adults in Asia. Several findings merit emphasis. First, absolute burden increased substantially, but aggregate Asia-wide changes in age-standardized rates were not statistically distinguishable from zero because the corresponding EAPC CIs crossed the null. This pattern underscores the divergence between demographic expansion and uncertain changes in underlying age-standardized risk. Second, substantial country heterogeneity persisted across socio-economic and epidemiological contexts. Third, sex- and age-related differences remained pronounced, reflecting unequal exposure to major risks and the increasing clinical complexity of cancer in ageing populations. Finally, decomposition and projection analyses indicate that demographic momentum will continue to shape future burden, even if age-specific risks decline.

The increasing burden of TBL cancer in older adults should be interpreted in the context of rapid population ageing across Asia. As more individuals survive into older age, the number entering age groups at the highest risk of lung cancer is expected to expand [18]. This dynamic helps explain why stable or declining age-standardized point estimates in some settings do not necessarily translate into a lower absolute population burden [19,20]. The current burden also reflects cumulative exposure to carcinogenic risks, especially tobacco smoking, whose effects often emerge after several decades [1]. In parts of Asia, this burden may be further reinforced by ambient air pollution, occupational carcinogens and other environmental exposures [3,21]. Together, these factors suggest that TBL cancer will remain a major challenge for health systems serving older adults, even in settings where prevention and treatment have improved [22].

The marked heterogeneity across countries indicates that TBL cancer burden in Asia is not determined by ageing alone, but by the interaction of historical exposure profiles, health-system capacity and stage of epidemiological transition [23]. Persistently high burden in Japan, China, the Republic of Korea and Türkiye may reflect earlier smoking epidemics, more complete ascertainment and longer survival into older age [5]. By contrast, lower recorded burdens in countries such as Nepal, Afghanistan, Kuwait and India should be interpreted cautiously, because they may reflect either lower cumulative risk exposure or incomplete diagnosis and registration [24]. The declines observed in several Central Asian countries suggest that reductions in age-standardized burden are achievable and may be associated with shifts in exposure patterns or broader improvements in public health [25]. Importantly, the attenuation of inequality across the SDI gradient should not be interpreted as uniform progress. It may reflect stabilization or reduction in some higher-SDI settings, but it may also reflect accumulating burden in lower- and middle-SDI countries as exposure profiles change and diagnostic transition remains incomplete. In this sense, convergence may partly represent epidemiological catch-up rather than equitable improvement across the region. This changing pattern underscores the need for country-specific cancer control strategies rather than a uniform regional response [3,23].

The sex- and age-specific patterns observed in this study are also epidemiologically informative. The greater burden in older men is likely to reflect the legacy of historically higher smoking prevalence in male populations across much of Asia [26], whereas the burden in women may be shaped relatively more by non-smoking determinants, including second-hand smoke and household or ambient air pollution [27,28]. These findings support the view that lung cancer causation in Asia is heterogeneous: tobacco remains dominant, but other exposures are important, especially among women and never-smokers [1,3]. Age-related gradients likely reflect both cumulative exposure to carcinogens and the biological consequences of ageing, including reduced immune surveillance, greater genomic instability and increasing multimorbidity [29,30]. These processes not only elevate cancer susceptibility but also complicate treatment and survival once disease occurs [31]. The findings therefore support sex-sensitive prevention and age-appropriate cancer control policies, including stronger tobacco control, reduction of environmental exposures and diagnostic pathways better adapted to older adults [22,31].

Our decomposition analysis provides further insight by showing that rising burden was not uniformly driven by worsening epidemiological risk. In many countries, the population-size and age-structure components accounted for much of the observed change, suggesting that demographic change increasingly shapes absolute burden [21,32]. At the same time, epidemiological contributions differed across settings, with some countries showing evidence of worsening age-specific burden and others showing improvement sufficient to offset part of the demographic pressure [6]. This distinction is important because it implies different policy priorities. Where demographic forces predominate, health systems will need to prepare for sustained growth in demand through expanded diagnostic capacity, geriatric oncology services and long-term care planning [22,33]. Where epidemiological deterioration is more evident, greater emphasis should be placed on upstream prevention, including tobacco control, environmental risk reduction and earlier detection [24,34].

The projection analyses reinforce this interpretation. Projected declines in ASRs, together with persistent absolute burden, suggest epidemiological improvement without proportional relief of clinical and service pressure [18,20]. This pattern is plausible because benefits from tobacco control, earlier diagnosis and therapeutic advances tend to accrue gradually, whereas population ageing continues to accelerate in many Asian countries [5,19]. The projected persistence of male predominance is also consistent with the long latency of smoking-related carcinogenesis, although a modest narrowing of the sex gap may indicate changing exposure profiles in later cohorts [8,26]. Importantly, stabilization or modest improvement in rates should not be interpreted as a diminishing public health challenge. Older adults with lung cancer frequently have multimorbidity and reduced physiological reserve, requiring more complex and resource-intensive care [2,33]. Future policy should combine primary prevention with age-appropriate diagnostic, treatment and supportive-care capacity for a rapidly growing older population [22,35].

These findings have differentiated policy implications. High-burden and rapidly ageing settings, such as Japan, China, the Republic of Korea and Türkiye, require integrated lung cancer control for older adults, combining tobacco control, risk-adapted early detection, timely diagnosis, treatment access and geriatric oncology capacity. In countries with low recorded rates but limited diagnostic and registration infrastructure, priorities should focus on strengthening cancer registries, pathology and imaging services, vital statistics and referral systems, because low estimated burden may partly reflect under-ascertainment. In Central Asian countries with declining ASRs, the priority is to sustain these gains through continued prevention, surveillance and equitable treatment access. Across the region, demographic momentum means that even modest age-specific risks can generate substantial absolute burden in large or rapidly ageing populations.

This study has several strengths. It specifically focuses on older adults, a population that bears a disproportionate and growing share of TBL cancer burden in Asia but has been insufficiently examined in previous regional analyses. By integrating trends, country comparisons, inequality metrics, decomposition analysis and BAPC projections, this study provides a broader assessment than descriptive burden estimates alone. It therefore offers not only a regional overview of current burden, but also insight into the demographic and epidemiological forces likely to shape future TBL cancer burden in ageing Asian societies.

This study has limitations. First, GBD estimates are modelled estimates and depend on the availability and quality of primary data. Cancer registry coverage, diagnostic capacity and cause-of-death certification vary widely across Asia, particularly in low-SDI settings, where sparse data and under-ascertainment may widen uncertainty intervals and bias burden estimates downwards. Therefore, low ASRs in some countries should not be interpreted as definitive evidence of low underlying risk. This uncertainty also affects country rankings, inequality estimates, and decomposition results. In particular, decomposition should be regarded as statistical attribution based on modelled age-specific rates, not causal inference; uncertainty in these rates may influence the estimated epidemiological-change component. Second, the ecological design precluded assessment of individual-level factors, including smoking, comorbidity, stage at diagnosis, histology, molecular subtype, screening and treatment access. Third, projections to 2050 extrapolated from historical trends and might not capture future changes in tobacco control, air pollution, screening uptake, therapeutic advances or health-system capacity. Nevertheless, the study provides a comparable regional assessment of the changing burden and structural drivers of TBL cancer in ageing Asian populations.

## Conclusion

In conclusion, TBL cancer remains a substantial and evolving health challenge among older adults in Asia. Although ASRs have declined in some settings, the overall burden continues to rise and is expected to remain high because of rapid population ageing and persistent exposure to major risk factors. Marked country heterogeneity and changing inequality patterns indicate that a uniform regional response is unlikely to be sufficient. These findings underscore the need for more targeted and age-responsive strategies in Asia, including strengthened tobacco control, reduction of environmental exposures, earlier diagnosis and expansion of geriatric cancer care to address the growing burden in ageing populations.

## Supplementary Material

Supplementary_material.docx

## Data Availability

All data used in this study are publicly available from the Global Health Data Exchange (GHDx) results tool at https://vizhub.healthdata.org/gbd-results/. The authors confirm that no additional, non-public data were used.

## Open scholarship

This article has earned the Center for Open Science badges for Open Data and Open Materials through Open Practices Disclosure. The data and materials are openly accessible at https://vizhub.healthdata.org/gbd-results/ and https://github.com/Z-C-Q/TBL-cancer.

## Abbreviations

Description
TBL: Tracheal, bronchus and lung
ASR: Age-standardized rate
ASIR: Age-standardized incidence rate
ASDR: Age-standardized death rate
ASPR: Age-standardized prevalence rate
BAPC: Bayesian age–period–cohort
CI: Confidence interval
CIX: Concentration index
DALY: Disability-adjusted life year
EAPC: Estimated annual percentage change
GBD: Global Burden of Disease
SDI: Socio-demographic Index
SII: Slope index of inequality
UI: Uncertainty interval
