# Supplementary material for: The shifting burden of tracheal, bronchus and lung cancer in ageing Asia: structural drivers and projections to 2050
Source: Ann Med. 2026 Sep 11;58(1):2722478. doi: 10.1080/07853890.2026.2722478 (PMC13573528; doi:10.1080/07853890.2026.2722478)
Supplement: Supplementary file 1 [file IANN_A_2722478_SM2434.docx]

**SUPPLEMENTAL**

**Methods**

Bayesian age–period–cohort model specification and diagnostics

1.1 Model inputs

1.2 Likelihood and linear predictor

1.3 Identifiability and overfitting control

1.4 Identifiability and overfitting control

1.5 Projection and age standardisation

1.6 Model diagnostics

1.7 Prior-sensitivity analysis

1.8 Uncertainty propagation

**Figure legend**

Figure S1. Prior-sensitivity analysis of Bayesian age–period–cohort projections for age-standardised rates of tracheal, bronchus, and lung cancer burden among older adults in Asia, 2024–2050.

Figure S2. Age‑standardised rates of tracheal, bronchus, and lung cancer among older adults in Asia, 2023. (A) Mortality. (B) DALYs. Abbreviations: ASR, Age‑standardised rate; DALYs, Disability-Adjusted Life Years.

Figure S3. Age‑specific number and rates of tracheal, bronchus, and lung cancer among older adults in Asia, 2023, by sex and age group. (A) Mortality. (B) DALYs. Abbreviations: DALYs, Disability-Adjusted Life Years.

Figure S4. Age‑specific trends of tracheal, bronchus, and lung cancer among older adults in Asia, 1990–2023. (A) Mortality. (B) DALYs. Abbreviations: ASR, Age‑standardised rate; DALYs, Disability-Adjusted Life Years.

Figure S5. Cross-country inequality in tracheal, bronchus, and lung cancer prevalence among older adults in Asia, 1990 and 2023. Abbreviations: SII, Slope index of inequality; CI, Concentration index; SDI, Socio-demographic Index.

Figure S6. Cross-country inequality in tracheal, bronchus, and lung cancer mortality among older adults in Asia, 1990 and 2023. Abbreviations: SII, Slope index of inequality; CI, Concentration index; SDI, Socio-demographic Index.

Figure S7. Cross-country inequality in tracheal, bronchus, and lung cancer DALYs among older adults in Asia, 1990 and 2023. Abbreviations: SII, Slope index of inequality; CI, Concentration index; SDI, Socio-demographic Index.

**Table legend**

Table S1. Country-level incidence and age-standardised incidence rates of tracheal, bronchus, and lung cancer among older adults in Asia in 1990 and 2023, with estimated annual percentage change.

Table S2. Country-level prevalence and age-standardised prevalence rates of tracheal, bronchus, and lung cancer among older adults in Asia in 1990 and 2023, with estimated annual percentage change.

Table S3. Country-level deaths and age-standardised death rates of tracheal, bronchus, and lung cancer among older adults in Asia in 1990 and 2023, with estimated annual percentage change.

Table S4. Country-level DALYs and age-standardised DALY rates of tracheal, bronchus, and lung cancer among older adults in Asia in 1990 and 2023, with estimated annual percentage change.

Table S5. Age‑specific incidence number and rates of tracheal, bronchus, and lung cancer among older adults in Asia, in 1990 and 2023, with EAPC, by sex and age group.

Table S6. Age‑specific prevalence number and rates of tracheal, bronchus, and lung cancer among older adults in Asia, in 1990 and 2023, with EAPC, by sex and age group.

Table S7. Age‑specific death number and rates of tracheal, bronchus, and lung cancer among older adults in Asia, in 1990 and 2023, with EAPC, by sex and age group.

Table S8. Age‑specific DALYs number and rates of tracheal, bronchus, and lung cancer among older adults in Asia, in 1990 and 2023, with EAPC, by sex and age group.

Table S9. Decomposition analysis of changes in tracheal, bronchus, and lung cancer incidence among older adults in Asia, 1990–2023, by country.

Table S10. Decomposition analysis of changes in tracheal, bronchus, and lung cancer prevalence among older adults in Asia, 1990–2023, by country.

Table S11. Decomposition analysis of changes in tracheal, bronchus, and lung cancer death among older adults in Asia, 1990–2023, by country.

Table S12. Decomposition analysis of changes in tracheal, bronchus, and lung cancer DALYs among older adults in Asia, 1990–2023, by country. Abbreviations: DALYs, Disability-Adjusted Life Years.

**Methods**

**Bayesian age–period–cohort model specification and diagnostics**

**1.1 Model inputs**

Bayesian age–period–cohort (BAPC) models were used to project the burden of tracheal, bronchus, and lung (TBL) cancer among adults aged 60 years and older in Asia to 2050. Models were fitted separately by sex and burden measure, including incidence, prevalence, deaths, and disability-adjusted life years (DALYs). The observed input data comprised GBD 2023 age-specific counts from 1990 to 2023 and corresponding age-, sex-, and year-specific population denominators. Five-year age groups from 60–64 years to 95 years and older were included. The period 1990–2023 was treated as the observed historical period, and 2024–2050 was treated as the projection period.

**1.2 Likelihood and linear predictor**

Let Yₐ,ₚ denote the observed number of events in age group a and calendar period p, and let Nₐ,ₚ denote the corresponding population. The observed counts were assumed to follow a Poisson distribution:

Y(a,p) ~ Poisson[N(a,p) × λ(a,p)]

where λₐ,ₚ represents the age- and period-specific event rate. The latent log rate was specified as:

ln[λ(a,p)] = μ + α(a) + β(p) + γ(c) + z(a,p), c = p − a

where μ is the intercept, αₐ is the age effect, βₚ is the period effect, γ꜀ is the cohort effect, c denotes birth cohort, and zₐ,ₚ is the overdispersion term. This formulation models age-specific counts while accounting for the population at risk.

**1.3 Prior specification and hyperparameters**

The final BAPC model used structured random-walk priors for the latent temporal components. Age and cohort effects were assigned second-order random-walk priors, period effects were assigned first-order random-walk priors, and the overdispersion component was modelled as an independent and identically distributed random effect. The prior structure was specified as follows:

αₐ–2×αₐ₋₁+αₐ₋₂ ~ Normal (0, τage⁻¹)

βₚ–βₚ₋₁ ~ Normal (0, τperiod⁻¹)

γ꜀–2×γ꜀₋₁+γ꜀₋₂ ~ Normal (0, τcohort⁻¹)

zₐ,ₚ ~ Normal (0, τoverdispersion⁻¹)

Precision parameters for the age, period, and cohort effects were assigned log-Gamma priors with hyperparameters 1 and 0.00005:

τage ~ log-Gamma (1, 0.00005)

τperiod ~ log-Gamma (1, 0.00005)

τcohort ~ log-Gamma (1, 0.00005)

The overdispersion precision was assigned a log-Gamma prior with hyperparameters 1 and 0.005:

τoverdispersion ~ log-Gamma (1, 0.005)

Thus, the final model specification used RW2 for age, RW1 for period, RW2 for cohort, and iid overdispersion.

**1.4 Identifiability and overfitting control**

Age, period, and cohort are linearly dependent, and APC models therefore require constraints or structured priors for stable estimation. In the BAPC-INLA framework, identifiability is handled through the latent Gaussian model structure and internal constraints applied to the random-walk components. The model estimates smoothed deviations rather than unconstrained independent fixed effects for age, period, and cohort. Accordingly, the age, period, and cohort components were used to improve projection and describe temporal structure, not to support causal interpretation of separately identifiable effects.

The random-walk priors also served as regularisation terms. RW1 priors penalise abrupt adjacent changes, whereas RW2 priors penalise excessive curvature. These smoothing constraints reduce overfitting to short-term fluctuations in the observed series while preserving long-term non-linear age, period, and cohort patterns supported by the data.

**1.5 Projection and age standardisation**

Future age-specific rates were generated from posterior predictive distributions estimated by integrated nested Laplace approximation. Projected event counts were obtained by applying projected age-specific rates to age-, sex-, and year-specific population forecasts. Age-standardised rates were calculated using the GBD world standard population weights restricted to the included older age groups, and reported per 100,000 population:

ASRₚ=Σₐ(wₐ×λₐ,ₚ)

where wₐ is the standard population weight for age group a, and λₐ,ₚ is the projected age-specific rate in age group a and period p. Rates were reported per 100,000 population.

Observed GBD estimates were available for 1990–2023, whereas values from 2024 onwards were model-derived projections. The observed 2023 GBD estimates were retained as empirical baselines for contextualising projected changes to 2050.

**1.6 Model diagnostics**

Model adequacy was assessed using retrospective prediction, posterior predictive checks, and prediction-interval review. The BAPC model was fitted with retrospective projection enabled, and posterior fitted trajectories during the observed period were visually compared with observed GBD estimates. The posterior fitted trajectories were reviewed to assess whether they captured the broad direction and curvature of the observed GBD series and whether there was evidence of systematic deviation during the historical period. Prediction intervals were reviewed to ensure that uncertainty widened appropriately over the long-term projection horizon. These checks were used to assess whether the fitted trajectories captured the broad temporal patterns in the observed data without excessive short-term fluctuation.

We assessed model fit by comparing the posterior fitted trajectories against the observed GBD data for the 1990–2023 period. We specifically examined whether the observed values fell within the 95% posterior predictive intervals, and evaluated whether the fitted curves captured the broad direction and curvature of the age-specific trends without systematic deviation. Prediction intervals for the projection horizon (2024–2050) were reviewed to confirm that uncertainty widened appropriately over time.

**1.7 Prior-sensitivity analysis**

To assess sensitivity to prior choice, we repeated the BAPC projections using two alternative prior specifications in addition to the baseline model used for the main analysis (Figure S1). The more flexible prior used log-Gamma (1, 0.0005) for the age, period, and cohort precision parameters and log-Gamma (1, 0.05) for the overdispersion precision. The smoother prior used log-Gamma (1, 0.000005) for the age, period, and cohort precision parameters and log-Gamma (1, 0.0005) for the overdispersion precision.

For overall Asia estimates in both sexes combined, the projected direction of decline in age-standardised rates from 2024 to 2050 was consistent across all three prior specifications. The projected percentage changes under the baseline, more flexible, and smoother priors were similar: -22.02%, -19.70%, and -22.42% for incidence; -8.38%, -4.07%, and -9.15% for prevalence; -25.47%, -23.85%, and -25.72% for deaths; and -25.67%, -23.38%, and -26.11% for DALYs, respectively. These findings support the robustness of our main projection conclusions. However, one sex-specific exception was observed for female prevalence, for which the projected direction was not fully consistent across prior specifications; therefore, subgroup-specific projections should be interpreted with caution.

**1.8 Uncertainty propagation**

Uncertainty was propagated through the Bayesian posterior estimated by INLA. For each future year, posterior distributions of age-specific rates were generated from the fitted model and combined with population projections to obtain posterior distributions of future event counts. Age-standardised rates were then calculated from posterior age-specific rates using standard population weights. Reported 95% prediction intervals represent uncertainty in the posterior predictive distribution of projected rates and counts. These prediction intervals should be interpreted as uncertainty in the BAPC projection framework, conditional on the observed GBD input estimates and population projection assumptions.

Figure S1. Prior-sensitivity analysis of Bayesian age–period–cohort projections for age-standardised rates of tracheal, bronchus, and lung cancer burden among older adults in Asia, 2024–2050.

Figure S2. Age‑standardised rates of tracheal, bronchus, and lung cancer among older adults in Asia, 2023. (A) Mortality. (B) DALYs. Abbreviations: ASR, Age‑standardised rate; DALYs, Disability-Adjusted Life Years.

Figure S3. Age‑specific number and rates of tracheal, bronchus, and lung cancer among older adults in Asia, 2023, by sex and age group. (A) Mortality. (B) DALYs. Abbreviations: DALYs, Disability-Adjusted Life Years.

Figure S4. Age‑specific trends of tracheal, bronchus, and lung cancer among older adults in Asia, 1990–2023. (A) Mortality. (B) DALYs. Abbreviations: ASR, Age‑standardised rate; DALYs, Disability-Adjusted Life Years.

Figure S5. Cross-country inequality in tracheal, bronchus, and lung cancer prevalence among older adults in Asia, 1990 and 2023. Abbreviations: SII, Slope index of inequality; CI, Concentration index; SDI, Socio-demographic Index.

Figure S6. Cross-country inequality in tracheal, bronchus, and lung cancer mortality among older adults in Asia, 1990 and 2023. Abbreviations: SII, Slope index of inequality; CI, Concentration index; SDI, Socio-demographic Index.

Figure S7. Cross-country inequality in tracheal, bronchus, and lung cancer DALYs among older adults in Asia, 1990 and 2023. Abbreviations: SII, Slope index of inequality; CI, Concentration index; DALYs=disability-adjusted life-years; SDI, Socio-demographic Index.

Table S1. Country-level incidence and age-standardised incidence rates of tracheal, bronchus, and lung cancer among older adults in Asia in 1990 and 2023, with estimated annual percentage change.

| **Country** | **1990** | | **2023** | | **EAPC**  **(95%CI)** |
| --- | --- | --- | --- | --- | --- |
|  | **Incident cases (95%UI)** | **ASRs of incidence per 100 000 (95%UI)** | **Incident cases (95%UI)** | **ASRs of incidence per 100 000 (95%UI)** |  |
| Afghanistan | 268 (176, 405) | 31.62 (20.85, 47.91) | 386 (264, 559) | 41.83 (28.70, 60.65) | 0.73 (-11.93, 15.21) |
| Armenia | 558 (506, 625) | 167.07 (151.42, 187.10) | 836 (743, 922) | 111.59 (99.12, 123.01) | -0.23 (-12.23, 13.42) |
| Azerbaijan | 658 (522, 790) | 114.61 (90.98, 137.53) | 1124 (857, 1730) | 75.80 (57.76, 116.60) | -0.44 (-12.56, 13.36) |
| Bahrain | 32 (22, 40) | 173.73 (123.06, 220.80) | 139 (102, 189) | 116.84 (86.14, 159.09) | -0.22 (-11.18, 12.09) |
| Bangladesh | 1938 (1337, 2669) | 35.06 (24.19, 48.29) | 10141 (6944, 14072) | 64.05 (43.85, 88.87) | 2.67 (-11.50, 19.12) |
| Bhutan | 6 (4, 9) | 21.59 (14.04, 32.32) | 25 (18, 35) | 31.69 (22.87, 45.53) | 2.14 (-9.37, 15.12) |
| Brunei Darussalam | 24 (18, 30) | 220.75 (167.42, 279.63) | 71 (58, 86) | 140.32 (114.77, 169.45) | 0.19 (-10.66, 12.35) |
| Cambodia | 238 (151, 353) | 48.54 (30.87, 72.21) | 1075 (774, 1478) | 63.44 (45.67, 87.21) | 1.94 (-10.79, 16.48) |
| China | 210952 (179018, 274738) | 208.47 (176.91, 271.51) | 701852 (578529, 817931) | 236.88 (195.26, 276.06) | 1.36 (-14.16, 19.68) |
| Cyprus | 186 (131, 252) | 184.36 (129.27, 249.60) | 454 (333, 575) | 181.07 (132.99, 229.52) | 0.90 (-10.92, 14.29) |
| Democratic People's Republic of Korea | 2047 (1528, 2680) | 115.02 (85.87, 150.63) | 6152 (4671, 8310) | 149.59 (113.58, 202.08) | 1.46 (-11.69, 16.58) |
| Georgia | 1004 (887, 1135) | 125.07 (110.44, 141.36) | 1105 (956, 1275) | 131.73 (114.00, 152.02) | 1.43 (-10.95, 15.54) |
| India | 15096 (11339, 21409) | 29.42 (22.10, 41.72) | 63641 (50159, 77509) | 41.46 (32.68, 50.49) | 2.30 (-13.35, 20.79) |
| Indonesia | 7270 (5041, 10216) | 66.31 (45.98, 93.18) | 27498 (19825, 35905) | 87.65 (63.19, 114.45) | 1.90 (-12.66, 18.88) |
| Iran (Islamic Republic of) | 2294 (1591, 3652) | 72.19 (50.08, 114.92) | 6323 (5243, 7764) | 64.06 (53.12, 78.66) | 0.71 (-12.77, 16.29) |
| Iraq | 913 (614, 1389) | 102.48 (68.98, 155.96) | 4269 (3150, 5467) | 152.79 (112.74, 195.64) | 2.45 (-10.58, 17.38) |
| Israel | 882 (821, 933) | 138.86 (129.20, 146.85) | 2398 (2093, 2660) | 147.46 (128.67, 163.54) | 0.62 (-12.05, 15.11) |
| Japan | 43778 (40456, 46283) | 201.48 (186.19, 213.00) | 128011 (107337, 140744) | 290.14 (243.28, 319.00) | 1.68 (-13.13, 19.03) |
| Jordan | 151 (103, 220) | 103.41 (70.37, 150.19) | 1004 (785, 1269) | 106.28 (83.11, 134.28) | 2.05 (-10.04, 15.78) |
| Kazakhstan | 3219 (3058, 3380) | 206.67 (196.32, 216.96) | 2168 (1982, 2366) | 79.60 (72.78, 86.89) | -2.43 (-14.78, 11.71) |
| Kuwait | 42 (37, 47) | 112.49 (100.40, 125.97) | 130 (110, 153) | 40.45 (34.30, 47.42) | -0.50 (-11.72, 12.14) |
| Kyrgyzstan | 428 (395, 464) | 118.33 (109.27, 128.33) | 385 (337, 433) | 58.98 (51.65, 66.35) | -1.85 (-13.43, 11.28) |
| Lao People's Democratic Republic | 175 (117, 252) | 71.29 (47.92, 102.72) | 654 (495, 850) | 114.01 (86.24, 148.03) | 2.31 (-10.06, 16.38) |
| Lebanon | 172 (121, 236) | 63.83 (45.07, 87.76) | 1240 (1001, 1496) | 166.92 (134.67, 201.36) | 4.46 (-8.19, 18.85) |
| Malaysia | 1201 (896, 1556) | 118.19 (88.20, 153.08) | 4672 (4008, 5652) | 114.63 (98.33, 138.67) | 0.59 (-12.31, 15.40) |
| Maldives | 6 (5, 9) | 63.84 (46.67, 90.23) | 39 (30, 49) | 106.23 (82.04, 132.44) | 2.83 (-8.32, 15.33) |
| Mongolia | 222 (139, 297) | 182.94 (114.67, 244.75) | 362 (285, 453) | 128.81 (101.51, 161.04) | -0.94 (-12.24, 11.80) |
| Myanmar | 2956 (2043, 4228) | 107.47 (74.29, 153.73) | 6783 (5099, 9479) | 110.51 (83.08, 154.43) | 0.95 (-12.39, 16.31) |
| Nepal | 300 (210, 443) | 28.58 (19.99, 42.26) | 1054 (784, 1432) | 33.07 (24.59, 44.93) | 0.67 (-12.32, 15.57) |
| Oman | 43 (31, 60) | 60.22 (43.60, 84.52) | 116 (87, 156) | 51.02 (38.22, 68.89) | 0.76 (-10.73, 13.74) |
| Pakistan | 2513 (1684, 3611) | 37.95 (25.43, 54.52) | 6159 (4582, 8682) | 38.70 (28.79, 54.55) | 0.68 (-13.33, 16.95) |
| Palestine | 85 (60, 119) | 81.26 (57.73, 113.98) | 351 (283, 445) | 111.47 (89.78, 141.10) | 1.97 (-9.94, 15.45) |
| Philippines | 3047 (2431, 3684) | 92.59 (73.85, 111.93) | 7419 (6298, 8879) | 71.76 (60.91, 85.88) | 0.21 (-13.31, 15.83) |
| Qatar | 9 (6, 13) | 105.27 (72.72, 153.39) | 43 (29, 65) | 48.72 (33.32, 73.51) | -1.50 (-12.16, 10.45) |
| Republic of Korea | 5057 (4303, 7448) | 150.11 (127.73, 221.12) | 30157 (24472, 35713) | 220.52 (178.94, 261.14) | 2.27 (-11.62, 18.35) |
| Saudi Arabia | 419 (322, 548) | 70.12 (53.94, 91.77) | 978 (759, 1433) | 64.49 (50.04, 94.52) | 0.73 (-11.84, 15.10) |
| Singapore | 657 (618, 691) | 257.47 (242.16, 270.65) | 1571 (1158, 2019) | 129.30 (95.33, 166.16) | -0.40 (-12.52, 13.41) |
| Sri Lanka | 708 (533, 970) | 58.36 (43.98, 79.97) | 3242 (2649, 4124) | 81.49 (66.58, 103.64) | 1.99 (-11.16, 17.09) |
| Syrian Arab Republic | 414 (287, 645) | 70.96 (49.20, 110.59) | 1254 (958, 1627) | 68.22 (52.13, 88.54) | 0.69 (-11.87, 15.05) |
| Taiwan (Province of China) | 3546 (3352, 3714) | 176.87 (167.23, 185.28) | 11106 (9890, 12413) | 184.18 (164.01, 205.85) | 0.37 (-12.75, 15.48) |
| Tajikistan | 274 (207, 326) | 85.44 (64.56, 101.62) | 364 (272, 580) | 52.04 (38.92, 82.97) | -1.70 (-13.31, 11.45) |
| Thailand | 6795 (5533, 8131) | 168.80 (137.45, 202.01) | 20010 (16319, 26759) | 129.05 (105.24, 172.57) | 0.01 (-13.77, 16.01) |
| Timor-Leste | 20 (13, 32) | 75.86 (47.07, 119.66) | 94 (67, 140) | 88.49 (62.98, 132.64) | 1.66 (-9.76, 14.51) |
| Turkmenistan | 172 (158, 186) | 77.71 (71.61, 84.32) | 225 (192, 262) | 42.85 (36.68, 49.85) | -1.26 (-12.83, 11.84) |
| Türkiye | 7810 (5712, 10687) | 195.21 (142.78, 267.13) | 26147 (18177, 32501) | 203.66 (141.59, 253.15) | 1.51 (-12.32, 17.52) |
| United Arab Emirates | 71 (49, 106) | 206.85 (141.10, 309.15) | 255 (183, 353) | 49.27 (35.38, 68.15) | -3.29 (-14.25, 9.07) |
| Uzbekistan | 1012 (928, 1104) | 84.21 (77.23, 91.89) | 1449 (1325, 1585) | 44.52 (40.69, 48.67) | -1.40 (-13.82, 12.82) |
| Viet Nam | 4570 (3507, 5914) | 90.57 (69.51, 117.22) | 16855 (12369, 21907) | 120.85 (88.68, 157.07) | 1.63 (-12.14, 17.56) |
| Yemen | 329 (224, 487) | 59.70 (40.58, 88.37) | 1005 (702, 1510) | 59.03 (41.19, 88.67) | 1.09 (-11.64, 15.65) |

Abbreviations: EAPC=estimated annual percentage change; UI=uncertainty interval; CI=confidence interval; ASR=age-standardised rate.

Table S2. Country-level prevalence and age-standardised prevalence rates of tracheal, bronchus, and lung cancer among older adults in Asia in 1990 and 2023, with estimated annual percentage change.

| **Country** | **1990** | | **2023** | | **EAPC**  **(95%CI)** |
| --- | --- | --- | --- | --- | --- |
|  | **Prevalent cases (95%UI)** | **ASRs of prevalence per 100 000 (95%UI)** | **Prevalent cases (95%UI)** | **ASRs of prevalence per 100 000 (95%UI)** |  |
| Afghanistan | 226 (151, 342) | 26.70 (17.89, 40.45) | 309 (221, 443) | 33.54 (24.01, 48.07) | 0.88 (-10.70, 13.97) |
| Armenia | 569 (521, 629) | 170.38 (155.81, 188.31) | 874 (790, 970) | 116.61 (105.41, 129.44) | -0.13 (-11.24, 12.37) |
| Azerbaijan | 646 (500, 780) | 112.57 (87.07, 135.91) | 1154 (871, 1795) | 77.81 (58.69, 121.01) | -0.29 (-11.66, 12.54) |
| Bahrain | 30 (21, 38) | 163.85 (116.41, 207.96) | 150 (113, 198) | 126.40 (95.16, 166.53) | 0.44 (-9.63, 11.64) |
| Bangladesh | 1850 (1337, 2456) | 33.46 (24.19, 44.43) | 10007 (6753, 13620) | 63.20 (42.65, 86.02) | 2.53 (-10.53, 17.48) |
| Bhutan | 6 (4, 9) | 19.98 (14.05, 31.78) | 22 (16, 31) | 28.80 (21.04, 40.05) | 2.01 (-8.29, 13.46) |
| Brunei Darussalam | 23 (18, 30) | 214.30 (164.16, 276.89) | 78 (62, 96) | 153.46 (121.03, 188.79) | 0.58 (-9.27, 11.50) |
| Cambodia | 226 (157, 327) | 46.15 (32.07, 66.90) | 989 (709, 1304) | 58.36 (41.86, 76.95) | 1.69 (-9.93, 14.82) |
| China | 208771 (174734, 270781) | 206.32 (172.68, 267.60) | 945059 (784273, 1183284) | 318.96 (264.70, 399.37) | 2.38 (-12.54, 19.85) |
| Cyprus | 176 (124, 248) | 173.75 (122.24, 245.47) | 593 (441, 802) | 236.49 (175.78, 319.91) | 2.35 (-8.52, 14.52) |
| Democratic People's Republic of Korea | 1997 (1515, 2630) | 112.22 (85.16, 147.83) | 6110 (4455, 8504) | 148.57 (108.34, 206.78) | 1.62 (-10.66, 15.60) |
| Georgia | 999 (881, 1125) | 124.39 (109.79, 140.11) | 1086 (952, 1242) | 129.47 (113.54, 148.11) | 1.58 (-10.01, 14.65) |
| India | 14461 (11022, 20800) | 28.18 (21.48, 40.54) | 60539 (49920, 75043) | 39.44 (32.52, 48.89) | 2.20 (-12.29, 19.08) |
| Indonesia | 6876 (4858, 9603) | 62.72 (44.31, 87.59) | 27074 (19983, 34637) | 86.30 (63.70, 110.40) | 1.89 (-11.52, 17.33) |
| Iran (Islamic Republic of) | 2241 (1561, 3581) | 70.52 (49.12, 112.68) | 7125 (5792, 9122) | 72.18 (58.68, 92.41) | 1.19 (-11.42, 15.60) |
| Iraq | 876 (604, 1318) | 98.35 (67.85, 147.99) | 4369 (3226, 5701) | 156.36 (115.45, 204.03) | 2.81 (-9.24, 16.46) |
| Israel | 889 (816, 962) | 139.94 (128.46, 151.45) | 3117 (2494, 3867) | 191.61 (153.33, 237.77) | 1.74 (-9.98, 14.98) |
| Japan | 68243 (58985, 78317) | 314.07 (271.46, 360.43) | 260989 (208299, 294268) | 591.54 (472.12, 666.97) | 2.85 (-11.10, 18.99) |
| Jordan | 149 (102, 216) | 102.13 (69.50, 147.39) | 1085 (833, 1357) | 114.82 (88.12, 143.63) | 2.55 (-8.69, 15.16) |
| Kazakhstan | 3154 (3003, 3331) | 202.47 (192.75, 213.82) | 2218 (2031, 2434) | 81.45 (74.58, 89.39) | -2.15 (-13.71, 10.95) |
| Kuwait | 46 (40, 53) | 124.03 (107.34, 141.99) | 197 (155, 255) | 61.26 (48.15, 79.39) | 0.64 (-9.77, 12.24) |
| Kyrgyzstan | 416 (388, 450) | 114.87 (107.18, 124.45) | 388 (343, 439) | 59.44 (52.54, 67.18) | -1.50 (-12.39, 10.73) |
| Lao People's Democratic Republic | 161 (108, 232) | 65.72 (43.90, 94.77) | 600 (455, 786) | 104.46 (79.19, 136.89) | 2.17 (-9.04, 14.76) |
| Lebanon | 170 (128, 236) | 63.18 (47.49, 87.74) | 1438 (1114, 1800) | 193.46 (149.96, 242.27) | 5.15 (-6.55, 18.32) |
| Malaysia | 1093 (828, 1377) | 107.56 (81.44, 135.46) | 4457 (3763, 5244) | 109.37 (92.34, 128.67) | 0.95 (-11.04, 14.56) |
| Maldives | 6 (5, 8) | 60.52 (45.15, 84.37) | 38 (30, 48) | 103.20 (81.23, 129.35) | 2.82 (-7.24, 13.98) |
| Mongolia | 207 (131, 276) | 170.79 (107.87, 227.51) | 363 (297, 454) | 129.07 (105.52, 161.45) | -0.63 (-11.15, 11.14) |
| Myanmar | 2773 (1923, 3972) | 100.85 (69.94, 144.42) | 6243 (4655, 8418) | 101.71 (75.84, 137.14) | 0.59 (-11.76, 14.65) |
| Nepal | 287 (201, 412) | 27.36 (19.20, 39.23) | 972 (722, 1277) | 30.51 (22.66, 40.06) | 0.51 (-11.38, 13.99) |
| Oman | 39 (28, 56) | 55.77 (39.12, 78.58) | 123 (91, 168) | 54.20 (40.01, 74.10) | 1.36 (-9.22, 13.18) |
| Pakistan | 2412 (1675, 3366) | 36.41 (25.29, 50.83) | 5820 (4231, 8073) | 36.57 (26.59, 50.73) | 0.60 (-12.28, 15.37) |
| Palestine | 79 (57, 113) | 75.79 (54.94, 107.82) | 388 (306, 486) | 123.08 (97.23, 154.36) | 2.42 (-8.47, 14.61) |
| Philippines | 2861 (2225, 3450) | 86.92 (67.62, 104.84) | 6950 (5935, 8572) | 67.22 (57.41, 82.92) | 0.27 (-12.24, 14.58) |
| Qatar | 9 (6, 13) | 107.01 (73.17, 150.63) | 60 (39, 94) | 67.97 (43.84, 106.36) | -0.13 (-10.01, 10.83) |
| Republic of Korea | 5307 (4511, 7529) | 157.54 (133.91, 223.51) | 59848 (41949, 77594) | 437.62 (306.74, 567.38) | 4.83 (-8.37, 19.92) |
| Saudi Arabia | 383 (293, 492) | 64.21 (49.04, 82.42) | 922 (702, 1370) | 60.84 (46.30, 90.37) | 0.94 (-10.67, 14.06) |
| Singapore | 716 (646, 806) | 280.35 (253.09, 315.93) | 3252 (2166, 4604) | 267.64 (178.24, 378.95) | 1.90 (-9.49, 14.71) |
| Sri Lanka | 662 (500, 894) | 54.61 (41.27, 73.76) | 3755 (2969, 4851) | 94.37 (74.63, 121.93) | 2.49 (-9.72, 16.35) |
| Syrian Arab Republic | 395 (278, 625) | 67.72 (47.70, 107.20) | 1325 (993, 1712) | 72.06 (54.05, 93.14) | 1.16 (-10.48, 14.30) |
| Taiwan (Province of China) | 3620 (3388, 3829) | 180.57 (169.01, 191.01) | 14404 (12155, 16866) | 238.86 (201.57, 279.70) | 1.56 (-10.80, 15.64) |
| Tajikistan | 270 (206, 319) | 84.42 (64.32, 99.54) | 351 (264, 557) | 50.25 (37.80, 79.65) | -1.62 (-12.48, 10.58) |
| Thailand | 6600 (5499, 7816) | 163.96 (136.61, 194.19) | 20520 (16413, 27338) | 132.34 (105.85, 176.31) | 0.27 (-12.43, 14.80) |
| Timor-Leste | 19 (12, 31) | 72.41 (46.44, 116.93) | 85 (62, 123) | 80.16 (58.41, 116.32) | 1.41 (-8.93, 12.93) |
| Turkmenistan | 170 (158, 184) | 76.85 (71.51, 83.43) | 227 (192, 265) | 43.30 (36.62, 50.55) | -1.01 (-11.82, 11.12) |
| Türkiye | 7682 (5516, 10689) | 192.03 (137.88, 267.18) | 28474 (19203, 37069) | 221.79 (149.58, 288.74) | 2.01 (-10.85, 16.72) |
| United Arab Emirates | 70 (47, 106) | 202.18 (137.84, 309.51) | 277 (200, 386) | 53.57 (38.61, 74.52) | -2.87 (-12.97, 8.39) |
| Uzbekistan | 1004 (930, 1086) | 83.58 (77.45, 90.39) | 1501 (1386, 1627) | 46.09 (42.56, 49.99) | -1.13 (-12.85, 12.16) |
| Viet Nam | 4421 (3258, 5647) | 87.63 (64.57, 111.92) | 17973 (13092, 23412) | 128.86 (93.86, 167.85) | 1.88 (-11.02, 16.64) |
| Yemen | 310 (212, 475) | 56.17 (38.41, 86.20) | 952 (661, 1358) | 55.91 (38.81, 79.72) | 1.13 (-10.46, 14.23) |

Abbreviations: EAPC=estimated annual percentage change; UI=uncertainty interval; CI=confidence interval; ASR=age-standardised rate.

Table S3. Country-level deaths and age-standardised death rates of tracheal, bronchus, and lung cancer among older adults in Asia in 1990 and 2023, with estimated annual percentage change.

| **Country** | **1990** | | **2023** | | **EAPC**  **(95%CI)** |
| --- | --- | --- | --- | --- | --- |
|  | **Deaths (95%UI)** | **ASRs of death per 100 000 (95%UI)** | **Deaths (95%UI)** | **ASRs of death per 100 000 (95%UI)** |  |
| Afghanistan | 289 (191, 435) | 34.17 (22.61, 51.44) | 431 (292, 620) | 46.72 (31.67, 67.30) | 1.13 (-8.07, 11.25) |
| Armenia | 569 (515, 633) | 170.10 (154.06, 189.44) | 867 (774, 949) | 115.69 (103.25, 126.64) | 0.29 (-8.17, 9.52) |
| Azerbaijan | 678 (537, 811) | 118.05 (93.56, 141.22) | 1138 (869, 1749) | 76.75 (58.58, 117.93) | -0.03 (-8.83, 9.62) |
| Bahrain | 33 (23, 42) | 180.65 (128.13, 229.42) | 133 (99, 180) | 112.56 (83.44, 151.63) | 0.66 (-6.66, 8.55) |
| Bangladesh | 2057 (1422, 2817) | 37.20 (25.73, 50.96) | 10436 (7149, 14535) | 65.90 (45.15, 91.79) | 2.75 (-7.88, 14.60) |
| Bhutan | 6 (4, 10) | 22.72 (14.75, 34.03) | 26 (19, 38) | 33.82 (24.51, 48.74) | 2.37 (-5.20, 10.54) |
| Brunei Darussalam | 25 (19, 32) | 231.23 (175.48, 293.84) | 71 (59, 86) | 140.15 (115.23, 168.45) | 0.85 (-6.14, 8.36) |
| Cambodia | 254 (161, 378) | 51.92 (32.98, 77.18) | 1156 (829, 1588) | 68.22 (48.91, 93.70) | 1.95 (-6.85, 11.59) |
| China | 215453 (183556, 281784) | 212.92 (181.40, 278.47) | 629734 (524804, 728854) | 212.54 (177.12, 245.99) | 1.58 (-10.76, 15.63) |
| Cyprus | 199 (139, 269) | 196.80 (137.96, 265.88) | 425 (313, 530) | 169.56 (124.83, 211.37) | 1.02 (-6.94, 9.66) |
| Democratic People's Republic of Korea | 2136 (1597, 2805) | 120.04 (89.74, 157.67) | 6333 (4795, 8524) | 153.98 (116.61, 207.26) | 1.53 (-8.03, 12.10) |
| Georgia | 1028 (910, 1161) | 128.07 (113.41, 144.65) | 1138 (987, 1312) | 135.72 (117.68, 156.46) | 1.64 (-7.37, 11.52) |
| India | 15662 (11715, 22130) | 30.52 (22.83, 43.13) | 66366 (52640, 80544) | 43.23 (34.29, 52.47) | 2.50 (-9.66, 16.29) |
| Indonesia | 7623 (5288, 10725) | 69.54 (48.24, 97.83) | 28299 (20442, 36792) | 90.20 (65.16, 117.27) | 1.87 (-8.90, 13.91) |
| Iran (Islamic Republic of) | 2370 (1650, 3766) | 74.57 (51.92, 118.51) | 6331 (5209, 7782) | 64.14 (52.77, 78.84) | 1.08 (-8.79, 12.02) |
| Iraq | 975 (653, 1485) | 109.43 (73.31, 166.73) | 4302 (3128, 5532) | 153.95 (111.94, 197.98) | 2.63 (-6.77, 12.98) |
| Israel | 915 (861, 957) | 144.11 (135.48, 150.59) | 2275 (1975, 2502) | 139.88 (121.44, 153.82) | 1.02 (-7.71, 10.57) |
| Japan | 36682 (34222, 38033) | 168.82 (157.50, 175.04) | 97117 (81293, 107534) | 220.12 (184.25, 243.73) | 1.34 (-9.62, 13.62) |
| Jordan | 157 (107, 228) | 107.31 (73.11, 155.52) | 983 (763, 1242) | 104.08 (80.72, 131.47) | 2.52 (-5.82, 11.60) |
| Kazakhstan | 3298 (3140, 3459) | 211.71 (201.57, 222.04) | 2187 (2011, 2375) | 80.32 (73.84, 87.22) | -1.94 (-11.08, 8.15) |
| Kuwait | 41 (37, 46) | 111.51 (100.10, 124.53) | 113 (97, 130) | 34.98 (30.04, 40.50) | 0.16 (-7.09, 7.97) |
| Kyrgyzstan | 443 (409, 479) | 122.44 (113.09, 132.52) | 391 (344, 439) | 59.92 (52.72, 67.17) | -1.38 (-9.74, 7.74) |
| Lao People's Democratic Republic | 187 (126, 271) | 76.54 (51.65, 110.60) | 695 (524, 902) | 121.04 (91.36, 157.05) | 2.49 (-6.33, 12.14) |
| Lebanon | 184 (130, 251) | 68.39 (48.37, 93.23) | 1251 (1011, 1516) | 168.35 (136.02, 203.97) | 4.21 (-4.14, 13.29) |
| Malaysia | 1298 (963, 1684) | 127.68 (94.80, 165.71) | 4927 (4240, 5956) | 120.89 (104.02, 146.13) | 1.00 (-8.30, 11.24) |
| Maldives | 7 (5, 9) | 67.27 (48.99, 94.83) | 41 (32, 51) | 111.27 (86.40, 139.41) | 2.99 (-4.30, 10.84) |
| Mongolia | 233 (146, 312) | 192.09 (120.15, 257.35) | 374 (296, 464) | 132.93 (105.17, 165.14) | -0.18 (-8.21, 8.55) |
| Myanmar | 3128 (2162, 4468) | 113.74 (78.60, 162.46) | 7156 (5371, 9944) | 116.58 (87.50, 162.00) | 0.98 (-9.10, 12.19) |
| Nepal | 314 (219, 463) | 29.93 (20.88, 44.13) | 1113 (827, 1519) | 34.92 (25.94, 47.66) | 0.97 (-8.27, 11.14) |
| Oman | 44 (32, 61) | 61.96 (44.80, 86.45) | 114 (85, 155) | 50.29 (37.46, 68.26) | 1.03 (-6.76, 9.47) |
| Pakistan | 2694 (1794, 3908) | 40.68 (27.08, 59.00) | 6539 (4806, 9157) | 41.09 (30.20, 57.54) | 0.72 (-9.64, 12.26) |
| Palestine | 91 (65, 128) | 86.91 (61.80, 122.34) | 352 (288, 442) | 111.61 (91.40, 140.28) | 2.10 (-5.96, 10.86) |
| Philippines | 3254 (2589, 3940) | 98.86 (78.67, 119.72) | 7807 (6632, 9331) | 75.51 (64.14, 90.25) | 0.12 (-9.79, 11.12) |
| Qatar | 9 (7, 14) | 108.18 (75.04, 156.72) | 39 (27, 58) | 43.74 (30.59, 65.98) | -0.23 (-6.98, 7.01) |
| Republic of Korea | 5045 (4229, 7686) | 149.77 (125.54, 228.17) | 22891 (19213, 26216) | 167.38 (140.49, 191.69) | 2.00 (-7.90, 12.96) |
| Saudi Arabia | 440 (339, 575) | 73.78 (56.73, 96.33) | 982 (762, 1403) | 64.76 (50.28, 92.55) | 0.89 (-7.96, 10.60) |
| Singapore | 649 (619, 673) | 254.13 (242.46, 263.69) | 1183 (919, 1445) | 97.35 (75.64, 118.96) | -0.39 (-8.41, 8.34) |
| Sri Lanka | 751 (563, 1035) | 61.91 (46.39, 85.34) | 3293 (2695, 4236) | 82.77 (67.73, 106.47) | 2.14 (-7.26, 12.50) |
| Syrian Arab Republic | 434 (302, 675) | 74.47 (51.88, 115.84) | 1280 (983, 1656) | 69.64 (53.50, 90.10) | 1.06 (-7.91, 10.89) |
| Taiwan (Province of China) | 3622 (3418, 3788) | 180.66 (170.50, 188.94) | 10411 (9380, 11411) | 172.65 (155.55, 189.24) | 0.81 (-8.59, 11.19) |
| Tajikistan | 284 (216, 339) | 88.51 (67.43, 105.79) | 374 (283, 592) | 53.55 (40.42, 84.67) | -1.37 (-9.68, 7.71) |
| Thailand | 7207 (5856, 8651) | 179.05 (145.49, 214.93) | 20910 (17080, 27894) | 134.85 (110.15, 179.90) | 0.40 (-9.54, 11.43) |
| Timor-Leste | 21 (13, 34) | 80.30 (50.00, 126.62) | 101 (72, 151) | 95.15 (68.12, 143.02) | 1.69 (-5.92, 9.91) |
| Turkmenistan | 177 (163, 192) | 79.98 (73.71, 86.79) | 229 (198, 265) | 43.63 (37.66, 50.52) | -0.88 (-9.11, 8.10) |
| Türkiye | 8077 (5902, 11077) | 201.90 (147.52, 276.88) | 26015 (18316, 32360) | 202.64 (142.66, 252.06) | 1.72 (-8.40, 12.97) |
| United Arab Emirates | 74 (50, 111) | 215.79 (146.06, 323.49) | 256 (183, 355) | 49.44 (35.37, 68.60) | -1.64 (-8.87, 6.17) |
| Uzbekistan | 1044 (958, 1136) | 86.88 (79.72, 94.55) | 1470 (1352, 1601) | 45.16 (41.52, 49.18) | -1.03 (-10.29, 9.18) |
| Viet Nam | 4761 (3653, 6163) | 94.36 (72.41, 122.15) | 16760 (12502, 21681) | 120.16 (89.63, 155.44) | 1.67 (-8.54, 13.01) |
| Yemen | 343 (234, 507) | 62.28 (42.35, 92.01) | 1064 (734, 1595) | 62.47 (43.10, 93.67) | 1.58 (-7.49, 11.54) |

Abbreviations: EAPC=estimated annual percentage change; UI=uncertainty interval; CI=confidence interval; ASR=age-standardised rate.

Table S4. Country-level DALYs and age-standardised DALY rates of tracheal, bronchus, and lung cancer among older adults in Asia in 1990 and 2023, with estimated annual percentage change.

| **Country** | **1990** | | **2023** | | **EAPC**  **(95%CI)** |
| --- | --- | --- | --- | --- | --- |
|  | **Number of DALYs (95%UI)** | **ASRs of DALYs per 100 000 (95%UI)** | **Number of DALYs (95%UI)** | **ASRs of DALYs per 100 000 (95%UI)** |  |
| Afghanistan | 5979 (3959, 9121) | 706.52 (467.75, 1077.76) | 7967 (5537, 11546) | 864.47 (600.76, 1252.76) | 0.92 (-10.96, 14.39) |
| Armenia | 13754 (12482, 15377) | 4114.98 (3734.39, 4600.85) | 18109 (16098, 19990) | 2416.36 (2147.99, 2667.32) | -0.21 (-11.30, 12.27) |
| Azerbaijan | 16176 (12658, 19370) | 2817.80 (2204.84, 3374.13) | 27012 (20281, 41828) | 1820.98 (1367.23, 2819.82) | -0.23 (-11.68, 12.70) |
| Bahrain | 723 (510, 915) | 3972.86 (2799.59, 5027.14) | 2995 (2176, 4088) | 2525.32 (1834.44, 3446.36) | 0.47 (-9.62, 11.70) |
| Bangladesh | 44859 (30943, 61761) | 811.41 (559.70, 1117.14) | 236314 (160380, 325713) | 1492.39 (1012.85, 2056.98) | 2.82 (-10.42, 18.01) |
| Bhutan | 144 (94, 217) | 503.45 (327.81, 759.03) | 549 (397, 777) | 704.77 (509.64, 998.52) | 2.23 (-8.14, 13.76) |
| Brunei Darussalam | 509 (387, 644) | 4709.30 (3581.86, 5957.11) | 1428 (1163, 1717) | 2805.17 (2284.80, 3372.17) | 0.66 (-9.08, 11.44) |
| Cambodia | 5441 (3465, 8104) | 1111.53 (707.92, 1655.66) | 23762 (17155, 32622) | 1402.05 (1012.23, 1924.87) | 1.86 (-9.70, 14.90) |
| China | 4755445 (4052706, 6191671) | 4699.56 (4005.08, 6118.91) | 12218028 (10132448, 14311070) | 4123.67 (3419.77, 4830.08) | 1.16 (-13.52, 18.33) |
| Cyprus | 3847 (2698, 5255) | 3805.84 (2668.90, 5199.02) | 7793 (5794, 9693) | 3109.35 (2311.76, 3867.62) | 0.89 (-9.68, 12.70) |
| Democratic People's Republic of Korea | 47154 (35291, 62167) | 2650.19 (1983.48, 3493.97) | 131283 (97971, 176731) | 3192.30 (2382.29, 4297.44) | 1.28 (-10.95, 15.19) |
| Georgia | 24182 (21268, 27509) | 3012.43 (2649.40, 3426.91) | 24726 (21283, 28688) | 2947.93 (2537.49, 3420.37) | 1.38 (-10.25, 14.52) |
| India | 363508 (274856, 521112) | 708.42 (535.65, 1015.56) | 1449619 (1123254, 1779922) | 944.33 (731.72, 1159.50) | 2.34 (-12.29, 19.41) |
| Indonesia | 172515 (119102, 242506) | 1573.59 (1086.39, 2212.02) | 651101 (469731, 856457) | 2075.35 (1497.24, 2729.91) | 1.92 (-11.48, 17.33) |
| Iran (Islamic Republic of) | 52125 (36019, 84600) | 1640.29 (1133.48, 2662.24) | 125540 (105283, 155650) | 1271.87 (1066.64, 1576.91) | 0.76 (-11.69, 14.96) |
| Iraq | 20459 (13801, 30918) | 2297.41 (1549.74, 3471.80) | 93158 (68082, 120377) | 3333.82 (2436.45, 4307.90) | 2.64 (-9.48, 16.38) |
| Israel | 18161 (17118, 19041) | 2858.96 (2694.83, 2997.53) | 41942 (37024, 46211) | 2578.71 (2276.36, 2841.21) | 0.78 (-10.55, 13.54) |
| Japan | 695985 (655788, 720091) | 3203.08 (3018.08, 3314.01) | 1451169 (1253340, 1576936) | 3289.12 (2840.73, 3574.17) | 0.74 (-12.59, 16.09) |
| Jordan | 3506 (2395, 5065) | 2396.36 (1637.03, 3461.39) | 21082 (16156, 26670) | 2231.28 (1709.91, 2822.68) | 2.22 (-8.91, 14.72) |
| Kazakhstan | 78637 (74265, 82325) | 5048.14 (4767.46, 5284.90) | 50253 (46177, 54529) | 1845.45 (1695.76, 2002.47) | -2.16 (-13.85, 11.13) |
| Kuwait | 914 (821, 1021) | 2472.20 (2221.03, 2760.15) | 2518 (2175, 2926) | 782.63 (675.92, 909.37) | -0.16 (-10.25, 11.06) |
| Kyrgyzstan | 10327 (9506, 11232) | 2854.31 (2627.57, 3104.57) | 8994 (7881, 10114) | 1377.09 (1206.70, 1548.59) | -1.54 (-12.52, 10.83) |
| Lao People's Democratic Republic | 3977 (2685, 5718) | 1624.06 (1096.51, 2334.77) | 14860 (11196, 19354) | 2588.71 (1950.37, 3371.57) | 2.48 (-9.04, 15.45) |
| Lebanon | 3817 (2671, 5303) | 1417.53 (992.06, 1969.66) | 24036 (19640, 29183) | 3234.58 (2642.98, 3927.11) | 4.07 (-7.17, 16.67) |
| Malaysia | 26556 (19764, 34358) | 2612.96 (1944.61, 3380.65) | 99343 (83635, 120708) | 2437.49 (2052.07, 2961.69) | 0.96 (-11.00, 14.53) |
| Maldives | 150 (110, 214) | 1499.23 (1101.56, 2140.78) | 769 (599, 970) | 2088.76 (1625.84, 2634.04) | 2.52 (-7.51, 13.63) |
| Mongolia | 5134 (3230, 6842) | 4234.04 (2663.89, 5642.25) | 8550 (6748, 10788) | 3039.75 (2399.33, 3835.62) | -0.25 (-11.01, 11.80) |
| Myanmar | 68523 (47275, 98346) | 2491.58 (1718.97, 3575.97) | 154034 (114591, 216215) | 2509.57 (1866.94, 3522.65) | 0.96 (-11.70, 15.43) |
| Nepal | 7131 (5007, 10630) | 679.80 (477.32, 1013.31) | 23874 (17725, 31941) | 748.91 (556.03, 1001.99) | 0.75 (-11.18, 14.28) |
| Oman | 989 (713, 1390) | 1397.08 (1007.46, 1962.83) | 2471 (1842, 3342) | 1089.96 (812.51, 1473.98) | 0.88 (-9.69, 12.69) |
| Pakistan | 57067 (38548, 79769) | 861.60 (582.01, 1204.36) | 140714 (105794, 201706) | 884.19 (664.77, 1267.45) | 0.80 (-12.14, 15.65) |
| Palestine | 1876 (1333, 2638) | 1797.42 (1276.88, 2527.36) | 7609 (6220, 9768) | 2415.22 (1974.43, 3100.77) | 2.12 (-8.72, 14.26) |
| Philippines | 69383 (55089, 83760) | 2108.19 (1673.87, 2545.02) | 167379 (142605, 201847) | 1618.94 (1379.32, 1952.32) | 0.18 (-12.31, 14.44) |
| Qatar | 213 (146, 312) | 2430.74 (1667.31, 3559.76) | 870 (597, 1324) | 984.15 (675.21, 1496.67) | -0.66 (-10.25, 9.95) |
| Republic of Korea | 111525 (94897, 159079) | 3310.73 (2817.14, 4722.44) | 386365 (328656, 443554) | 2825.16 (2403.19, 3243.33) | 1.16 (-11.30, 15.36) |
| Saudi Arabia | 9555 (7306, 12475) | 1600.84 (1224.03, 2090.00) | 20394 (15718, 30521) | 1345.23 (1036.80, 2013.27) | 0.73 (-10.85, 13.80) |
| Singapore | 13333 (12725, 13892) | 5223.19 (4984.77, 5442.06) | 21141 (16568, 25965) | 1740.09 (1363.64, 2137.14) | -0.92 (-11.63, 11.08) |
| Sri Lanka | 16140 (12203, 21831) | 1331.02 (1006.33, 1800.36) | 68497 (54328, 88158) | 1721.62 (1365.50, 2215.78) | 2.07 (-10.00, 15.75) |
| Syrian Arab Republic | 9499 (6574, 15002) | 1629.21 (1127.67, 2573.26) | 26585 (20183, 34889) | 1446.39 (1098.11, 1898.21) | 0.85 (-10.78, 14.00) |
| Taiwan (Province of China) | 77714 (73622, 81342) | 3876.77 (3672.64, 4057.75) | 186743 (170510, 205155) | 3096.85 (2827.65, 3402.17) | 0.34 (-11.65, 13.95) |
| Tajikistan | 6622 (4927, 7886) | 2066.88 (1537.91, 2461.49) | 8574 (6373, 13770) | 1226.30 (911.47, 1969.50) | -1.61 (-12.55, 10.70) |
| Thailand | 153388 (125382, 182728) | 3810.66 (3114.90, 4539.57) | 406937 (329360, 552740) | 2624.43 (2124.12, 3564.74) | 0.06 (-12.42, 14.33) |
| Timor-Leste | 470 (291, 740) | 1760.39 (1089.53, 2772.98) | 2022 (1430, 3065) | 1912.07 (1352.18, 2898.77) | 1.51 (-8.91, 13.12) |
| Turkmenistan | 4194 (3850, 4551) | 1897.03 (1741.52, 2058.34) | 5457 (4644, 6405) | 1039.75 (884.81, 1220.45) | -0.94 (-11.88, 11.35) |
| Türkiye | 186418 (136615, 255357) | 4659.76 (3414.85, 6382.96) | 536706 (368206, 682764) | 4180.52 (2868.03, 5318.19) | 1.43 (-11.26, 15.95) |
| United Arab Emirates | 1647 (1131, 2456) | 4791.58 (3290.17, 7144.54) | 5614 (4061, 7797) | 1084.66 (784.66, 1506.47) | -2.11 (-12.12, 9.03) |
| Uzbekistan | 24510 (22458, 26814) | 2040.23 (1869.44, 2232.05) | 34509 (31660, 37402) | 1059.95 (972.42, 1148.79) | -1.14 (-12.96, 12.29) |
| Viet Nam | 107327 (80113, 139503) | 2127.18 (1587.81, 2764.90) | 383896 (274694, 503949) | 2752.42 (1969.48, 3613.17) | 1.72 (-11.07, 16.34) |
| Yemen | 7715 (5212, 11506) | 1398.87 (945.13, 2086.23) | 22238 (15411, 33707) | 1305.78 (904.92, 1979.19) | 1.36 (-10.41, 14.68) |

Abbreviations: DALYs=disability-adjusted life-years; EAPC=estimated annual percentage change; UI=uncertainty interval; CI=confidence interval; ASR=age-standardised rate.

Table S5. Age‑specific incidence number and rates of tracheal, bronchus, and lung cancer among older adults in Asia, in 1990 and 2023, with EAPC, by sex and age group.

| **Age** | **Sex** | **1990** | | **2023** | | **EAPC**  **(95%CI)** |
| --- | --- | --- | --- | --- | --- | --- |
|  |  | **Incident cases (95%UI)** | **Age-specific rate per 100 000 (95%UI)** | **Incident cases (95%UI)** | **Age-specific rate per 100 000 (95%UI)** |  |
| 60-64 years | Both | 87035 (71092, 112357) | 101.54 (82.94, 131.08) | 182542 (135372, 237510) | 89.36 (66.27, 116.26) | 1.01 (-7.67, 10.50) |
|  | Female | 22302 (17020, 29899) | 53.03 (40.47, 71.09) | 49518 (34623, 67789) | 47.91 (33.50, 65.59) | 0.90 (-6.96, 9.43) |
|  | Male | 64733 (50137, 89416) | 148.27 (114.84, 204.81) | 133024 (90444, 183830) | 131.79 (89.61, 182.13) | 1.07 (-6.80, 9.62) |
| 65-69 years | Both | 89000 (72282, 118028) | 141.49 (114.92, 187.64) | 235435 (177196, 300466) | 136.12 (102.45, 173.72) | 1.26 (-7.16, 10.45) |
|  | Female | 23674 (18402, 31341) | 73.72 (57.30, 97.60) | 62943 (44279, 85262) | 71.27 (50.14, 96.55) | 1.12 (-6.50, 9.36) |
|  | Male | 65325 (51083, 95605) | 212.19 (165.93, 310.54) | 172493 (124062, 232994) | 203.78 (146.57, 275.26) | 1.33 (-6.27, 9.54) |
| 70-74 years | Both | 74996 (61930, 98503) | 176.08 (145.41, 231.28) | 244495 (194176, 306832) | 185.13 (147.03, 232.33) | 1.50 (-6.56, 10.25) |
|  | Female | 22153 (17076, 29513) | 97.67 (75.28, 130.12) | 69955 (49801, 93398) | 101.40 (72.19, 135.38) | 1.23 (-6.05, 9.07) |
|  | Male | 52842 (40790, 77715) | 265.43 (204.89, 390.36) | 174540 (130049, 232500) | 276.71 (206.18, 368.60) | 1.58 (-5.61, 9.33) |
| 75-79 years | Both | 48490 (39879, 64005) | 180.83 (148.72, 238.70) | 185737 (143185, 227882) | 223.28 (172.12, 273.94) | 2.28 (-5.34, 10.51) |
|  | Female | 15764 (12191, 20602) | 105.68 (81.73, 138.11) | 58004 (40841, 76550) | 130.08 (91.59, 171.68) | 1.97 (-4.91, 9.33) |
|  | Male | 32726 (25449, 48063) | 275.05 (213.89, 403.95) | 127734 (90959, 166234) | 330.93 (235.65, 430.67) | 2.37 (-4.32, 9.53) |
| 80-84 years | Both | 23893 (19328, 30768) | 167.66 (135.62, 215.90) | 133175 (97884, 164867) | 262.51 (192.95, 324.98) | 3.38 (-3.64, 10.91) |
|  | Female | 8775 (6838, 11833) | 104.50 (81.43, 140.91) | 47722 (33515, 64020) | 167.64 (117.73, 224.89) | 3.15 (-3.18, 9.90) |
|  | Male | 15118 (11625, 21661) | 258.27 (198.59, 370.04) | 85453 (56765, 112476) | 383.81 (254.96, 505.18) | 3.43 (-2.56, 9.79) |
| 85-89 years | Both | 9168 (7332, 11566) | 165.11 (132.05, 208.32) | 82453 (59795, 102245) | 305.20 (221.33, 378.46) | 4.52 (-1.60, 11.02) |
|  | Female | 3744 (2778, 4944) | 108.15 (80.26, 142.83) | 33650 (22827, 46214) | 206.95 (140.39, 284.23) | 4.36 (-1.15, 10.18) |
|  | Male | 5424 (4100, 7352) | 259.42 (196.12, 351.67) | 48804 (34031, 65170) | 453.70 (316.37, 605.86) | 4.55 (-0.40, 9.75) |
| 90-94 years | Both | 1720 (1318, 2171) | 123.29 (94.45, 155.61) | 31470 (21925, 39582) | 304.57 (212.20, 383.09) | 5.87 (1.07, 10.89) |
|  | Female | 847 (590, 1142) | 91.35 (63.57, 123.14) | 15620 (9950, 21506) | 234.30 (149.25, 322.59) | 5.84 (1.57, 10.30) |
|  | Male | 873 (663, 1182) | 186.62 (141.72, 252.66) | 15850 (10493, 20996) | 432.36 (286.23, 572.74) | 5.86 (2.41, 9.43) |
| 95+ years | Both | 261 (188, 346) | 87.34 (62.90, 115.69) | 7486 (5135, 9856) | 241.83 (165.88, 318.35) | 6.64 (3.48, 9.90) |
|  | Female | 145 (96, 207) | 69.98 (46.40, 99.59) | 4575 (2903, 6371) | 210.23 (133.37, 292.73) | 6.88 (4.13, 9.70) |
|  | Male | 116 (81, 172) | 126.59 (88.10, 186.97) | 2911 (2007, 4050) | 316.62 (218.31, 440.49) | 6.33 (4.66, 8.03) |

Abbreviations: EAPC=estimated annual percentage change; UI=uncertainty interval; CI=confidence interval.

Table S6. Age‑specific prevalence number and rates of tracheal, bronchus, and lung cancer among older adults in Asia, in 1990 and 2023, with EAPC, by sex and age group.

| **Age** | **Sex** | **1990** | | **2023** | | **EAPC**  **(95%CI)** |
| --- | --- | --- | --- | --- | --- | --- |
|  |  | **Prevalent cases (95%UI)** | **Age-specific rate per 100 000 (95%UI)** | **Prevalent cases (95%UI)** | **Age-specific rate per 100 000 (95%UI)** |  |
| 60-64 years | Both | 107471 (87436, 135310) | 125.38 (102.01, 157.86) | 286930 (221779, 372927) | 140.46 (108.56, 182.55) | 1.85 (-6.89, 11.43) |
|  | Female | 27899 (20800, 36127) | 66.34 (49.46, 85.90) | 83576 (60548, 112125) | 80.87 (58.59, 108.49) | 1.91 (-6.03, 10.52) |
|  | Male | 79573 (61856, 106479) | 182.26 (141.68, 243.89) | 203354 (148476, 274724) | 201.47 (147.10, 272.18) | 1.86 (-6.08, 10.47) |
| 65-69 years | Both | 100337 (82348, 132786) | 159.52 (130.92, 211.11) | 360601 (275563, 459608) | 208.49 (159.33, 265.74) | 2.35 (-6.16, 11.63) |
|  | Female | 27012 (21176, 34773) | 84.12 (65.94, 108.28) | 101790 (71912, 136863) | 115.26 (81.43, 154.98) | 2.33 (-5.38, 10.67) |
|  | Male | 73325 (58559, 101821) | 238.17 (190.21, 330.73) | 258811 (191137, 339543) | 305.76 (225.81, 401.13) | 2.36 (-5.31, 10.66) |
| 70-74 years | Both | 76812 (62635, 99161) | 180.35 (147.06, 232.82) | 366607 (280036, 462677) | 277.59 (212.04, 350.34) | 2.81 (-5.36, 11.67) |
|  | Female | 23382 (18026, 30747) | 103.09 (79.47, 135.55) | 109430 (77682, 149416) | 158.62 (112.60, 216.58) | 2.56 (-4.81, 10.51) |
|  | Male | 53429 (41510, 76614) | 268.37 (208.51, 384.83) | 257177 (181961, 338222) | 407.72 (288.48, 536.21) | 2.89 (-4.40, 10.73) |
| 75-79 years | Both | 43168 (35175, 56713) | 160.99 (131.18, 211.50) | 241331 (179820, 301214) | 290.10 (216.16, 362.09) | 3.66 (-4.06, 12.00) |
|  | Female | 14140 (10934, 18260) | 94.79 (73.30, 122.41) | 77083 (54576, 102590) | 172.87 (122.40, 230.08) | 3.37 (-3.60, 10.84) |
|  | Male | 29028 (22569, 40512) | 243.97 (189.68, 340.49) | 164248 (117553, 212006) | 425.53 (304.55, 549.26) | 3.75 (-3.03, 11.01) |
| 80-84 years | Both | 18718 (15072, 24395) | 131.34 (105.76, 171.18) | 148248 (106258, 193125) | 292.22 (209.45, 380.68) | 4.74 (-2.38, 12.37) |
|  | Female | 6876 (5273, 9114) | 81.89 (62.80, 108.53) | 53972 (36225, 73549) | 189.60 (127.25, 258.37) | 4.59 (-1.83, 11.44) |
|  | Male | 11841 (9259, 16466) | 202.29 (158.16, 281.30) | 94276 (62996, 127794) | 423.44 (282.94, 573.98) | 4.74 (-1.32, 11.18) |
| 85-89 years | Both | 6961 (5425, 8967) | 125.37 (97.70, 161.50) | 82624 (57810, 108656) | 305.83 (213.98, 402.19) | 5.49 (-0.69, 12.05) |
|  | Female | 2800 (2037, 3705) | 80.87 (58.85, 107.03) | 35145 (22230, 49018) | 216.15 (136.72, 301.47) | 5.51 (-0.06, 11.39) |
|  | Male | 4161 (3146, 5653) | 199.03 (150.47, 270.38) | 47479 (30391, 66146) | 441.39 (282.53, 614.93) | 5.41 (0.42, 10.65) |
| 90-94 years | Both | 990 (747, 1260) | 70.97 (53.50, 90.32) | 24463 (15878, 32267) | 236.76 (153.67, 312.29) | 7.03 (2.18, 12.11) |
|  | Female | 498 (343, 687) | 53.69 (36.93, 74.07) | 13609 (8066, 19082) | 204.13 (120.99, 286.24) | 7.36 (3.03, 11.88) |
|  | Male | 492 (373, 655) | 105.24 (79.68, 140.08) | 10854 (7286, 15039) | 296.08 (198.76, 410.24) | 6.62 (3.14, 10.21) |
| 95+ years | Both | 138 (99, 183) | 46.25 (33.03, 61.22) | 4046 (2752, 5383) | 130.70 (88.89, 173.86) | 6.75 (3.59, 10.01) |
|  | Female | 76 (51, 107) | 36.61 (24.42, 51.59) | 2484 (1563, 3417) | 114.12 (71.82, 157.01) | 7.05 (4.30, 9.87) |
|  | Male | 62 (43, 89) | 68.04 (46.87, 97.43) | 1562 (1083, 2110) | 169.93 (117.83, 229.46) | 6.36 (4.68, 8.06) |

Abbreviations: EAPC=estimated annual percentage change; UI=uncertainty interval; CI=confidence interval.

Table S7. Age‑specific death number and rates of tracheal, bronchus, and lung cancer among older adults in Asia, in 1990 and 2023, with EAPC, by sex and age group.

| **Age** | **Sex** | **1990** | | **2023** | | **EAPC**  **(95%CI)** |
| --- | --- | --- | --- | --- | --- | --- |
|  |  | **Deaths (95%UI)** | **Age-specific rate per 100 000 (95%UI)** | **Deaths (95%UI)** | **Age-specific rate per 100 000 (95%UI)** |  |
| 60-64 years | Both | 80862 (66032, 104488) | 94.34 (77.04, 121.90) | 154243 (114013, 200229) | 75.50 (55.81, 98.02) | 0.68 (-7.97, 10.14) |
|  | Female | 20618 (15752, 27662) | 49.03 (37.45, 65.77) | 40499 (28177, 55667) | 39.19 (27.26, 53.86) | 0.49 (-7.34, 8.99) |
|  | Male | 60245 (46643, 83744) | 137.99 (106.84, 191.82) | 113744 (77163, 156813) | 112.69 (76.45, 155.36) | 0.78 (-7.08, 9.29) |
| 65-69 years | Both | 85908 (69733, 114021) | 136.58 (110.86, 181.27) | 201426 (153057, 255600) | 116.46 (88.49, 147.78) | 0.85 (-7.54, 10.00) |
|  | Female | 22710 (17504, 30167) | 70.72 (54.51, 93.94) | 52140 (36656, 71431) | 59.04 (41.51, 80.89) | 0.62 (-6.96, 8.83) |
|  | Male | 63198 (49291, 92984) | 205.28 (160.11, 302.03) | 149286 (107180, 200050) | 176.36 (126.62, 236.34) | 0.94 (-6.63, 9.13) |
| 70-74 years | Both | 74892 (61713, 98707) | 175.84 (144.90, 231.76) | 210120 (165636, 260991) | 159.10 (125.42, 197.62) | 1.00 (-7.02, 9.71) |
|  | Female | 22009 (16991, 29429) | 97.03 (74.91, 129.75) | 59080 (42174, 78833) | 85.64 (61.13, 114.27) | 0.69 (-6.55, 8.49) |
|  | Male | 52883 (40662, 78254) | 265.63 (204.24, 393.07) | 151040 (111351, 201103) | 239.45 (176.53, 318.82) | 1.10 (-6.06, 8.81) |
| 75-79 years | Both | 51988 (42810, 69280) | 193.88 (159.65, 258.36) | 169520 (128972, 208018) | 203.78 (155.04, 250.06) | 1.72 (-5.85, 9.91) |
|  | Female | 16922 (13048, 22352) | 113.45 (87.47, 149.85) | 52430 (37718, 70244) | 117.58 (84.59, 157.54) | 1.38 (-5.45, 8.70) |
|  | Male | 35065 (27203, 52019) | 294.71 (228.63, 437.20) | 117090 (82825, 152913) | 303.36 (214.58, 396.16) | 1.83 (-4.82, 8.95) |
| 80-84 years | Both | 27700 (22258, 36200) | 194.38 (156.18, 254.02) | 130497 (98335, 160312) | 257.23 (193.84, 316.00) | 2.77 (-4.21, 10.26) |
|  | Female | 10231 (7883, 13879) | 121.84 (93.88, 165.28) | 46646 (33579, 63127) | 163.86 (117.96, 221.76) | 2.51 (-3.78, 9.22) |
|  | Male | 17469 (13242, 25551) | 298.43 (226.21, 436.49) | 83851 (55720, 111002) | 376.61 (250.26, 498.56) | 2.84 (-3.11, 9.17) |
| 85-89 years | Both | 11006 (8683, 14150) | 198.22 (156.39, 254.85) | 84104 (63108, 104486) | 311.31 (233.59, 386.75) | 3.94 (-2.15, 10.40) |
|  | Female | 4559 (3362, 6087) | 131.71 (97.12, 175.85) | 34239 (23486, 46285) | 210.58 (144.44, 284.66) | 3.74 (-1.74, 9.53) |
|  | Male | 6446 (4802, 8956) | 308.32 (229.67, 428.38) | 49865 (35081, 65759) | 463.57 (326.13, 611.33) | 4.00 (-0.92, 9.17) |
| 90-94 years | Both | 2445 (1847, 3109) | 175.21 (132.38, 222.83) | 36026 (26046, 44879) | 348.66 (252.08, 434.35) | 5.08 (0.32, 10.07) |
|  | Female | 1195 (824, 1620) | 128.86 (88.79, 174.69) | 17304 (11334, 23800) | 259.57 (170.01, 357.01) | 4.96 (0.72, 9.37) |
|  | Male | 1249 (931, 1715) | 267.13 (198.93, 366.61) | 18722 (12438, 24445) | 510.69 (339.28, 666.81) | 5.17 (1.74, 8.71) |
| 95+ years | Both | 464 (335, 616) | 154.99 (111.87, 205.69) | 11943 (8211, 15630) | 385.78 (265.23, 504.88) | 6.27 (3.12, 9.52) |
|  | Female | 255 (168, 365) | 122.93 (81.20, 175.80) | 7082 (4521, 9889) | 325.43 (207.74, 454.39) | 6.42 (3.69, 9.23) |
|  | Male | 209 (145, 310) | 227.46 (157.67, 337.49) | 4861 (3372, 6798) | 528.62 (366.72, 739.33) | 6.10 (4.42, 7.80) |

Abbreviations: EAPC=estimated annual percentage change; UI=uncertainty interval; CI=confidence interval.

Table S8. Age‑specific DALYs number and rates of tracheal, bronchus, and lung cancer among older adults in Asia, in 1990 and 2023, with EAPC, by sex and age group.

| **Age** | **Sex** | **1990** | | **2023** | | **EAPC**  **(95%CI)** |
| --- | --- | --- | --- | --- | --- | --- |
|  |  | **Number of DALYs (95%UI)** | **Age-specific rate per 100 000 (95%UI)** | **Number of DALYs (95%UI)** | **Age-specific rate per 100 000 (95%UI)** |  |
| 60-64 years | Both | 2354478 (1923239, 3043024) | 2746.91 (2243.80, 3550.22) | 4505766 (3336262, 5841192) | 2205.64 (1633.15, 2859.35) | 0.69 (-7.96, 10.15) |
|  | Female | 599988 (459288, 803866) | 1426.65 (1092.10, 1911.43) | 1183114 (826839, 1623932) | 1144.78 (800.05, 1571.32) | 0.50 (-7.33, 8.99) |
|  | Male | 1754490 (1359262, 2438781) | 4018.71 (3113.43, 5586.10) | 3322652 (2262296, 4571335) | 3291.87 (2241.33, 4528.98) | 0.78 (-7.08, 9.30) |
| 65-69 years | Both | 2110048 (1716783, 2798757) | 3354.63 (2729.40, 4449.57) | 4934205 (3766128, 6253760) | 2852.86 (2177.50, 3615.81) | 0.86 (-7.53, 10.01) |
|  | Female | 557037 (430112, 739601) | 1734.62 (1339.37, 2303.13) | 1276235 (897143, 1745833) | 1445.17 (1015.90, 1976.93) | 0.63 (-6.95, 8.84) |
|  | Male | 1553011 (1213119, 2285265) | 5044.42 (3940.40, 7422.90) | 3657970 (2638948, 4892421) | 4321.49 (3117.63, 5779.86) | 0.95 (-6.62, 9.13) |
| 70-74 years | Both | 1513564 (1250076, 1995554) | 3553.75 (2935.10, 4685.43) | 4253099 (3354896, 5282878) | 3220.42 (2540.31, 4000.17) | 1.00 (-7.02, 9.72) |
|  | Female | 444089 (343043, 593144) | 1957.88 (1512.39, 2615.03) | 1194824 (854991, 1588063) | 1731.89 (1239.30, 2301.88) | 0.69 (-6.54, 8.50) |
|  | Male | 1069475 (823338, 1585384) | 5371.96 (4135.61, 7963.36) | 3058275 (2258632, 4064045) | 4848.50 (3580.77, 6443.01) | 1.11 (-6.06, 8.82) |
| 75-79 years | Both | 842141 (693572, 1121488) | 3140.59 (2586.54, 4182.36) | 2749753 (2091653, 3370769) | 3305.49 (2514.38, 4052.01) | 1.71 (-5.86, 9.90) |
|  | Female | 273504 (211308, 360990) | 1833.57 (1416.61, 2420.08) | 848153 (612848, 1133839) | 1902.15 (1374.43, 2542.86) | 1.37 (-5.46, 8.69) |
|  | Male | 568637 (442150, 843874) | 4779.16 (3716.09, 7092.42) | 1901600 (1351584, 2476327) | 4926.63 (3501.66, 6415.62) | 1.82 (-4.83, 8.94) |
| 80-84 years | Both | 352275 (283107, 460466) | 2471.93 (1986.58, 3231.11) | 1651968 (1246420, 2027182) | 3256.32 (2456.91, 3995.93) | 2.76 (-4.22, 10.24) |
|  | Female | 129737 (100048, 175789) | 1544.99 (1191.43, 2093.40) | 588808 (424888, 794364) | 2068.42 (1492.58, 2790.51) | 2.50 (-3.80, 9.20) |
|  | Male | 222538 (168438, 325658) | 3801.66 (2877.45, 5563.29) | 1063160 (708393, 1405210) | 4775.12 (3181.71, 6311.42) | 2.83 (-3.13, 9.15) |
| 85-89 years | Both | 111495 (87948, 143360) | 2008.06 (1583.98, 2581.97) | 847432 (635667, 1051787) | 3136.75 (2352.90, 3893.16) | 3.93 (-2.16, 10.39) |
|  | Female | 46088 (33923, 61551) | 1331.38 (979.96, 1778.10) | 344048 (236238, 463200) | 2115.97 (1452.92, 2848.78) | 3.73 (-1.75, 9.51) |
|  | Male | 65407 (48696, 90727) | 3128.46 (2329.18, 4339.53) | 503384 (354653, 661824) | 4679.73 (3297.04, 6152.66) | 3.99 (-0.93, 9.16) |
| 90-94 years | Both | 21411 (16191, 27199) | 1534.47 (1160.38, 1949.33) | 316240 (228715, 393855) | 3060.63 (2213.55, 3811.81) | 5.09 (0.33, 10.08) |
|  | Female | 10468 (7187, 14197) | 1128.48 (774.81, 1530.55) | 151993 (99613, 208858) | 2279.93 (1494.21, 3132.91) | 4.97 (0.73, 9.38) |
|  | Male | 10943 (8150, 15012) | 2339.59 (1742.48, 3209.46) | 164247 (109297, 213469) | 4480.37 (2981.44, 5823.06) | 5.18 (1.75, 8.72) |
| 95+ years | Both | 3810 (2754, 5050) | 1273.03 (920.18, 1687.54) | 97064 (66722, 126811) | 3135.33 (2155.23, 4096.21) | 6.23 (3.08, 9.48) |
|  | Female | 2089 (1379, 2986) | 1006.81 (664.59, 1439.14) | 57414 (36659, 80190) | 2638.08 (1684.42, 3684.63) | 6.39 (3.66, 9.19) |
|  | Male | 1721 (1195, 2549) | 1874.75 (1302.17, 2777.29) | 39650 (27567, 55485) | 4312.28 (2998.11, 6034.48) | 6.06 (4.39, 7.76) |

Abbreviations: DALYs=disability-adjusted life-years; EAPC=estimated annual percentage change; UI=uncertainty interval; CI=confidence interval.

Table S9. Decomposition analysis of changes in tracheal, bronchus, and lung cancer incidence among older adults in Asia, 1990–2023, by country.

| **Country** | **Overall Difference** | **Aging** | **Aging Percentage** | **Population** | **Population Percentage** | **Epidemiological Change** | **Epidemiological Change Percentage** |
| --- | --- | --- | --- | --- | --- | --- | --- |
| Afghanistan | 117.98 | 43.07 | 36.51 | 27.5 | 23.31 | 47.42 | 40.19 |
| Armenia | 277.89 | 10.95 | 3.94 | 575.85 | 207.23 | -308.91 | -111.17 |
| Azerbaijan | 466.52 | 24.56 | 5.27 | 870.26 | 186.54 | -428.3 | -91.81 |
| Bahrain | 106.95 | -5.3 | -4.96 | 145.97 | 136.49 | -33.73 | -31.54 |
| Bangladesh | 8202.9 | -4.91 | -0.06 | 5105.92 | 62.25 | 3101.89 | 37.81 |
| Bhutan | 18.5 | 0.37 | 1.98 | 13.13 | 70.97 | 5 | 27.04 |
| Brunei Darussalam | 47.57 | -3.02 | -6.34 | 72.7 | 152.84 | -22.12 | -46.49 |
| Cambodia | 837.66 | 20.66 | 2.47 | 674.03 | 80.47 | 142.96 | 17.07 |
| China | 490900.35 | 23687.23 | 4.83 | 430499.76 | 87.7 | 36713.36 | 7.48 |
| Cyprus | 267.52 | -7.69 | -2.87 | 273.64 | 102.29 | 1.57 | 0.59 |
| Democratic People's Republic of Korea | 4105.08 | 112.75 | 2.75 | 3080.37 | 75.04 | 911.97 | 22.22 |
| Georgia | 100.87 | -7.24 | -7.18 | 46.11 | 45.71 | 62 | 61.47 |
| India | 48544.41 | 400.36 | 0.82 | 36142.84 | 74.45 | 12001.21 | 24.72 |
| Indonesia | 20228.65 | 83.36 | 0.41 | 15710.35 | 77.66 | 4434.93 | 21.92 |
| Iran (Islamic Republic of) | 4028.84 | 240.14 | 5.96 | 4538.88 | 112.66 | -750.18 | -18.62 |
| Iraq | 3356.69 | -65.44 | -1.95 | 2439.54 | 72.68 | 982.59 | 29.27 |
| Israel | 1516.55 | 40.65 | 2.68 | 1416.11 | 93.38 | 59.79 | 3.94 |
| Japan | 84231.86 | 18475.79 | 21.93 | 53905.26 | 64 | 11850.81 | 14.07 |
| Jordan | 852.84 | -2.2 | -0.26 | 838.01 | 98.26 | 17.02 | 2 |
| Kazakhstan | -1051.76 | 54.88 | -5.22 | 1669.62 | -158.75 | -2776.26 | 263.96 |
| Kuwait | 88.58 | -8.87 | -10.01 | 218.21 | 246.35 | -120.77 | -136.34 |
| Kyrgyzstan | -42.9 | 8.3 | -19.36 | 259.75 | -605.48 | -310.95 | 724.83 |
| Lao People's Democratic Republic | 479.91 | -5.06 | -1.05 | 305.24 | 63.6 | 179.73 | 37.45 |
| Lebanon | 1068.57 | 30.25 | 2.83 | 543.88 | 50.9 | 494.44 | 46.27 |
| Malaysia | 3470.76 | 60.49 | 1.74 | 3565.22 | 102.72 | -154.94 | -4.46 |
| Maldives | 32.73 | 0.89 | 2.7 | 22.61 | 69.08 | 9.24 | 28.21 |
| Mongolia | 140.48 | -14.78 | -10.52 | 248.52 | 176.9 | -93.26 | -66.38 |
| Myanmar | 3827.13 | -20.68 | -0.54 | 3691.83 | 96.46 | 155.98 | 4.08 |
| Nepal | 754.53 | 14.65 | 1.94 | 658.29 | 87.25 | 81.59 | 10.81 |
| Oman | 73.04 | -3.15 | -4.32 | 86.78 | 118.82 | -10.59 | -14.5 |
| Pakistan | 3645.34 | 4.92 | 0.13 | 3560.63 | 97.68 | 79.79 | 2.19 |
| Palestine | 266.36 | -4.69 | -1.76 | 203.12 | 76.26 | 67.93 | 25.5 |
| Philippines | 4372.24 | -40.21 | -0.92 | 5791.45 | 132.46 | -1379 | -31.54 |
| Qatar | 33.86 | -2.17 | -6.42 | 61.23 | 180.84 | -25.2 | -74.43 |
| Republic of Korea | 25100.41 | 1529.83 | 6.09 | 18576.32 | 74.01 | 4994.26 | 19.9 |
| Saudi Arabia | 605.43 | -43.33 | -7.16 | 669.64 | 110.61 | -20.88 | -3.45 |
| Singapore | 913.62 | 51.47 | 5.63 | 1851.75 | 202.68 | -989.59 | -108.32 |
| Sri Lanka | 2536.43 | 42.39 | 1.67 | 1938.17 | 76.41 | 555.87 | 21.92 |
| Syrian Arab Republic | 840.12 | 2.77 | 0.33 | 874.39 | 104.08 | -37.05 | -4.41 |
| Taiwan (Province of China) | 7560.92 | 668.42 | 8.84 | 7185.51 | 95.03 | -293.01 | -3.88 |
| Tajikistan | 90.12 | 0.69 | 0.76 | 263 | 291.85 | -173.58 | -192.61 |
| Thailand | 13215.74 | 503.92 | 3.81 | 17091.53 | 129.33 | -4379.72 | -33.14 |
| Timor-Leste | 73.33 | 7.22 | 9.84 | 65.32 | 89.08 | 0.79 | 1.08 |
| Turkmenistan | 53.04 | 3.07 | 5.79 | 183.4 | 345.8 | -133.43 | -251.58 |
| Türkiye | 18337.02 | 364.31 | 1.99 | 17535.24 | 95.63 | 437.48 | 2.39 |
| United Arab Emirates | 183.91 | -48.42 | -26.33 | 625.34 | 340.03 | -393.01 | -213.7 |
| Uzbekistan | 437.03 | 30.03 | 6.87 | 1323.61 | 302.87 | -916.62 | -209.74 |
| Viet Nam | 12285.72 | -141.44 | -1.15 | 9410.37 | 76.6 | 3016.79 | 24.56 |
| Yemen | 676.1 | 20.58 | 3.04 | 681.7 | 100.83 | -26.17 | -3.87 |

Table S10. Decomposition analysis of changes in tracheal, bronchus, and lung cancer prevalence among older adults in Asia, 1990–2023, by country.

| **Country** | **Overall Difference** | **Aging** | **Aging Percentage** | **Population** | **Population Percentage** | **Epidemiological Change** | **Epidemiological Change Percentage** |
| --- | --- | --- | --- | --- | --- | --- | --- |
| Afghanistan | 83.22 | 19.14 | 23 | 22.6 | 27.15 | 41.49 | 49.85 |
| Armenia | 304.47 | -6.36 | -2.09 | 592.01 | 194.44 | -281.17 | -92.35 |
| Azerbaijan | 508.04 | 42.95 | 8.45 | 870.43 | 171.33 | -405.34 | -79.78 |
| Bahrain | 120.08 | -0.96 | -0.8 | 145.95 | 121.54 | -24.9 | -20.74 |
| Bangladesh | 8157.27 | 101.58 | 1.25 | 4968.45 | 60.91 | 3087.24 | 37.85 |
| Bhutan | 16.71 | -0.31 | -1.86 | 12.06 | 72.18 | 4.96 | 29.69 |
| Brunei Darussalam | 54.96 | -1.16 | -2.11 | 74.08 | 134.8 | -17.96 | -32.68 |
| Cambodia | 763.13 | 3.66 | 0.48 | 629.72 | 82.52 | 129.75 | 17 |
| China | 736287.6 | 7188.62 | 0.98 | 508893.29 | 69.12 | 220205.69 | 29.91 |
| Cyprus | 417.1 | -6.24 | -1.5 | 307.54 | 73.73 | 115.79 | 27.76 |
| Democratic People's Republic of Korea | 4113.13 | -19.33 | -0.47 | 3040.56 | 73.92 | 1091.9 | 26.55 |
| Georgia | 87.43 | -31.46 | -35.99 | 45.55 | 52.1 | 73.34 | 83.89 |
| India | 46077.84 | -558.06 | -1.21 | 34560.01 | 75 | 12075.89 | 26.21 |
| Indonesia | 20198.48 | 108.17 | 0.54 | 15204.38 | 75.27 | 4885.92 | 24.19 |
| Iran (Islamic Republic of) | 4883.49 | 78.53 | 1.61 | 4760.01 | 97.47 | 44.95 | 0.92 |
| Iraq | 3493.32 | 14.7 | 0.42 | 2426.37 | 69.46 | 1052.24 | 30.12 |
| Israel | 2227.86 | -17.79 | -0.8 | 1643.89 | 73.79 | 601.75 | 27.01 |
| Japan | 192744.67 | 17028.52 | 8.83 | 99186.64 | 51.46 | 76529.51 | 39.71 |
| Jordan | 935.45 | -2.01 | -0.22 | 867.14 | 92.7 | 70.32 | 7.52 |
| Kazakhstan | -935.97 | 59.38 | -6.34 | 1655.8 | -176.91 | -2651.14 | 283.25 |
| Kuwait | 151.27 | -3.52 | -2.33 | 265.72 | 175.66 | -110.93 | -73.33 |
| Kyrgyzstan | -27.37 | 18.04 | -65.89 | 255.69 | -934.1 | -301.1 | 1099.99 |
| Lao People's Democratic Republic | 438.7 | -3.2 | -0.73 | 280.23 | 63.88 | 161.68 | 36.85 |
| Lebanon | 1267.5 | 0.69 | 0.05 | 608.93 | 48.04 | 657.89 | 51.9 |
| Malaysia | 3364.24 | 33.71 | 1 | 3321.32 | 98.72 | 9.21 | 0.27 |
| Maldives | 31.95 | 0.63 | 1.96 | 21.79 | 68.2 | 9.54 | 29.84 |
| Mongolia | 155.93 | -5.01 | -3.21 | 239.42 | 153.55 | -78.49 | -50.34 |
| Myanmar | 3469.6 | -65.95 | -1.9 | 3431.03 | 98.89 | 104.52 | 3.01 |
| Nepal | 685.48 | -2.78 | -0.41 | 618.25 | 90.19 | 70.01 | 10.21 |
| Oman | 83.4 | -2.02 | -2.42 | 85.88 | 102.97 | -0.46 | -0.55 |
| Pakistan | 3408.6 | -16.53 | -0.48 | 3390.65 | 99.47 | 34.48 | 1.01 |
| Palestine | 308.63 | -0.65 | -0.21 | 209.38 | 67.84 | 99.9 | 32.37 |
| Philippines | 4089.41 | -6.16 | -0.15 | 5432.35 | 132.84 | -1336.77 | -32.69 |
| Qatar | 50.73 | -1.22 | -2.4 | 69.85 | 137.68 | -17.9 | -35.28 |
| Republic of Korea | 54541.05 | 1350.35 | 2.48 | 30069.48 | 55.13 | 23121.21 | 42.39 |
| Saudi Arabia | 583.24 | -19.33 | -3.31 | 621.77 | 106.61 | -19.2 | -3.29 |
| Singapore | 2536.01 | 27.64 | 1.09 | 2619.33 | 103.29 | -110.97 | -4.38 |
| Sri Lanka | 3094.18 | -30.16 | -0.97 | 2069.86 | 66.9 | 1054.48 | 34.08 |
| Syrian Arab Republic | 929.72 | 3.14 | 0.34 | 877.25 | 94.36 | 49.33 | 5.31 |
| Taiwan (Province of China) | 10783.85 | 256.71 | 2.38 | 8370.29 | 77.62 | 2156.86 | 20 |
| Tajikistan | 80.88 | 11.92 | 14.74 | 257.99 | 318.97 | -189.03 | -233.71 |
| Thailand | 13920.07 | 76.47 | 0.55 | 16975.94 | 121.95 | -3132.34 | -22.5 |
| Timor-Leste | 65.45 | 3.47 | 5.3 | 60.63 | 92.64 | 1.35 | 2.06 |
| Turkmenistan | 57.34 | 4.9 | 8.54 | 182.88 | 318.95 | -130.43 | -227.49 |
| Türkiye | 20791.34 | -74.48 | -0.36 | 18207.58 | 87.57 | 2658.24 | 12.79 |
| United Arab Emirates | 207.75 | -19.36 | -9.32 | 626.39 | 301.51 | -399.28 | -192.19 |
| Uzbekistan | 495.79 | 48.6 | 9.8 | 1334.32 | 269.13 | -887.13 | -178.93 |
| Viet Nam | 13552.12 | -73.49 | -0.54 | 9635.02 | 71.1 | 3990.59 | 29.45 |
| Yemen | 642.44 | 4.77 | 0.74 | 644.01 | 100.24 | -6.34 | -0.99 |

Table S11. Decomposition analysis of changes in tracheal, bronchus, and lung cancer death among older adults in Asia, 1990–2023, by country.

| **Country** | **Overall Difference** | **Aging** | **Aging Percentage** | **Population** | **Population Percentage** | **Epidemiological Change** | **Epidemiological Change Percentage** |
| --- | --- | --- | --- | --- | --- | --- | --- |
| Afghanistan | 141.35 | 57.68 | 40.81 | 30.25 | 21.4 | 53.42 | 37.79 |
| Armenia | 298.49 | 20.99 | 7.03 | 591.13 | 198.04 | -313.63 | -105.07 |
| Azerbaijan | 460.72 | 11.96 | 2.6 | 889.91 | 193.15 | -441.15 | -95.75 |
| Bahrain | 100.61 | -6.77 | -6.73 | 147.1 | 146.21 | -39.72 | -39.48 |
| Bangladesh | 8378.91 | -68.41 | -0.82 | 5318.26 | 63.47 | 3129.05 | 37.34 |
| Bhutan | 19.84 | 0.92 | 4.65 | 13.9 | 70.08 | 5.01 | 25.27 |
| Brunei Darussalam | 46.35 | -4.09 | -8.83 | 74.71 | 161.19 | -24.27 | -52.37 |
| Cambodia | 901.96 | 34.32 | 3.8 | 722.6 | 80.12 | 145.04 | 16.08 |
| China | 414281.34 | 31686.27 | 7.65 | 411751.86 | 99.39 | -29156.8 | -7.04 |
| Cyprus | 226.13 | -7.4 | -3.27 | 274.05 | 121.19 | -40.52 | -17.92 |
| Democratic People's Republic of Korea | 4196.78 | 202.9 | 4.83 | 3187.97 | 75.96 | 805.92 | 19.2 |
| Georgia | 110.28 | 7.43 | 6.73 | 47.39 | 42.98 | 55.46 | 50.29 |
| India | 50703.87 | 1000.93 | 1.97 | 37564.54 | 74.09 | 12138.4 | 23.94 |
| Indonesia | 20675.62 | 57.95 | 0.28 | 16301.01 | 78.84 | 4316.67 | 20.88 |
| Iran (Islamic Republic of) | 3961.26 | 364.31 | 9.2 | 4618.8 | 116.6 | -1021.84 | -25.8 |
| Iraq | 3327.23 | -120.87 | -3.63 | 2521.68 | 75.79 | 926.41 | 27.84 |
| Israel | 1360.02 | 81.78 | 6.01 | 1403.95 | 103.23 | -125.71 | -9.24 |
| Japan | 60434.24 | 18733.59 | 31 | 42738.89 | 70.72 | -1038.25 | -1.72 |
| Jordan | 826.39 | -2.76 | -0.33 | 844.78 | 102.23 | -15.63 | -1.89 |
| Kazakhstan | -1110.75 | 47.29 | -4.26 | 1702.77 | -153.3 | -2860.8 | 257.56 |
| Kuwait | 71.32 | -11.68 | -16.38 | 208.11 | 291.81 | -125.12 | -175.43 |
| Kyrgyzstan | -51.63 | 1.76 | -3.41 | 266.84 | -516.86 | -320.22 | 620.28 |
| Lao People's Democratic Republic | 507.39 | -5.56 | -1.1 | 325.47 | 64.15 | 187.48 | 36.95 |
| Lebanon | 1066.9 | 45.44 | 4.26 | 556.42 | 52.15 | 465.03 | 43.59 |
| Malaysia | 3629.5 | 76.31 | 2.1 | 3806.6 | 104.88 | -253.42 | -6.98 |
| Maldives | 34.25 | 1.01 | 2.94 | 23.74 | 69.32 | 9.5 | 27.74 |
| Mongolia | 140.97 | -19.52 | -13.85 | 258.95 | 183.69 | -98.45 | -69.84 |
| Myanmar | 4027.58 | 26.45 | 0.66 | 3900.63 | 96.85 | 100.51 | 2.5 |
| Nepal | 799.3 | 25.31 | 3.17 | 692.18 | 86.6 | 81.81 | 10.24 |
| Oman | 70.13 | -3.46 | -4.93 | 87.51 | 124.77 | -13.91 | -19.83 |
| Pakistan | 3844.82 | 37.15 | 0.97 | 3799.19 | 98.81 | 8.48 | 0.22 |
| Palestine | 260.9 | -7.07 | -2.71 | 209.27 | 80.21 | 58.7 | 22.5 |
| Philippines | 4553.57 | -51.64 | -1.13 | 6144.31 | 134.93 | -1539.1 | -33.8 |
| Qatar | 29.2 | -2.67 | -9.13 | 60.25 | 206.31 | -28.38 | -97.18 |
| Republic of Korea | 17845.45 | 1571.63 | 8.81 | 15896.11 | 89.08 | 377.71 | 2.12 |
| Saudi Arabia | 586.74 | -51.65 | -8.8 | 688.37 | 117.32 | -49.98 | -8.52 |
| Singapore | 534 | 67.02 | 12.55 | 1687.49 | 316.01 | -1220.51 | -228.56 |
| Sri Lanka | 2544.49 | 78.03 | 3.07 | 2004.85 | 78.79 | 461.61 | 18.14 |
| Syrian Arab Republic | 845.85 | 0.69 | 0.08 | 905.99 | 107.11 | -60.83 | -7.19 |
| Taiwan (Province of China) | 6789.38 | 945.8 | 13.93 | 7039.06 | 103.68 | -1195.48 | -17.61 |
| Tajikistan | 90.84 | -6.35 | -6.99 | 271.61 | 298.99 | -174.42 | -192 |
| Thailand | 13702.81 | 864 | 6.31 | 18034.33 | 131.61 | -5195.51 | -37.92 |
| Timor-Leste | 79.2 | 9.46 | 11.94 | 69.74 | 88.06 | 0 | 0 |
| Turkmenistan | 52.12 | 1.82 | 3.49 | 187.94 | 360.63 | -137.65 | -264.12 |
| Türkiye | 17937.73 | 594.82 | 3.32 | 17785.26 | 99.15 | -442.35 | -2.47 |
| United Arab Emirates | 181.68 | -64.25 | -35.36 | 644.86 | 354.94 | -398.93 | -219.58 |
| Uzbekistan | 425.83 | 17.28 | 4.06 | 1356.36 | 318.52 | -947.81 | -222.58 |
| Viet Nam | 11998.71 | -154.2 | -1.29 | 9547.26 | 79.57 | 2605.66 | 21.72 |
| Yemen | 720.51 | 33.06 | 4.59 | 716.01 | 99.38 | -28.56 | -3.96 |

Table S12. Decomposition analysis of changes in tracheal, bronchus, and lung cancer DALYs among older adults in Asia, 1990–2023, by country. Abbreviations: DALYs, Disability-Adjusted Life Years.

| **Country** | **Overall Difference** | **Aging** | **Aging Percentage** | **Population** | **Population Percentage** | **Epidemiological Change** | **Epidemiological Change Percentage** |
| --- | --- | --- | --- | --- | --- | --- | --- |
| Afghanistan | 1987.79 | 530.92 | 26.71 | 589.33 | 29.65 | 867.54 | 43.64 |
| Armenia | 4355.87 | -302.29 | -6.94 | 13462.21 | 309.06 | -8804.05 | -202.12 |
| Azerbaijan | 10835.44 | 1028.61 | 9.49 | 21215.75 | 195.8 | -11408.92 | -105.29 |
| Bahrain | 2271.82 | -51.51 | -2.27 | 3266.89 | 143.8 | -943.57 | -41.53 |
| Bangladesh | 191455.06 | 2343.68 | 1.22 | 118454.04 | 61.87 | 70657.33 | 36.91 |
| Bhutan | 404.77 | -8.03 | -1.98 | 298.77 | 73.81 | 114.02 | 28.17 |
| Brunei Darussalam | 918.89 | -26.04 | -2.83 | 1513.83 | 164.75 | -568.91 | -61.91 |
| Cambodia | 18321.01 | 84.27 | 0.46 | 15148.66 | 82.68 | 3088.09 | 16.86 |
| China | 7462583.07 | 47906.13 | 0.64 | 8555533.1 | 114.65 | -1140856.16 | -15.29 |
| Cyprus | 3946.48 | -85 | -2.15 | 5178.14 | 131.21 | -1146.67 | -29.06 |
| Democratic People's Republic of Korea | 84129.02 | -350.95 | -0.42 | 68127.33 | 80.98 | 16352.64 | 19.44 |
| Georgia | 543.93 | -831.74 | -152.91 | 1069.43 | 196.61 | 306.24 | 56.3 |
| India | 1086110.97 | -14167.23 | -1.3 | 844350.53 | 77.74 | 255927.67 | 23.56 |
| Indonesia | 478586.42 | 2411.68 | 0.5 | 372347.58 | 77.8 | 103827.16 | 21.69 |
| Iran (Islamic Republic of) | 73416.47 | 1601.09 | 2.18 | 97142.46 | 132.32 | -25327.08 | -34.5 |
| Iraq | 72698.36 | 240.69 | 0.33 | 53665.57 | 73.82 | 18792.11 | 25.85 |
| Israel | 23785.33 | -17.06 | -0.07 | 26901.55 | 113.1 | -3099.15 | -13.03 |
| Japan | 755177.26 | 131080.89 | 17.36 | 716692.52 | 94.9 | -92596.16 | -12.26 |
| Jordan | 17575.45 | -40.08 | -0.23 | 18496.03 | 105.24 | -880.49 | -5.01 |
| Kazakhstan | -28384.56 | 1205.22 | -4.25 | 40195.35 | -141.61 | -69785.14 | 245.86 |
| Kuwait | 1604.34 | -51.88 | -3.23 | 4664.72 | 290.76 | -3008.5 | -187.52 |
| Kyrgyzstan | -1332.65 | 433.03 | -32.49 | 6210.91 | -466.06 | -7976.6 | 598.55 |
| Lao People's Democratic Republic | 10883.18 | -72.72 | -0.67 | 6936.2 | 63.73 | 4019.71 | 36.94 |
| Lebanon | 20219.59 | 30.6 | 0.15 | 11041.62 | 54.61 | 9147.37 | 45.24 |
| Malaysia | 72787.19 | 718.64 | 0.99 | 77346 | 106.26 | -5277.45 | -7.25 |
| Maldives | 619.26 | 12.97 | 2.09 | 477.76 | 77.15 | 128.53 | 20.76 |
| Mongolia | 3415.57 | -87 | -2.55 | 5805.84 | 169.98 | -2303.28 | -67.43 |
| Myanmar | 85511.24 | -1578.09 | -1.85 | 84709.82 | 99.06 | 2379.51 | 2.78 |
| Nepal | 16742.61 | -108.3 | -0.65 | 15268.31 | 91.19 | 1582.61 | 9.45 |
| Oman | 1481.81 | -39.96 | -2.7 | 1940.84 | 130.98 | -419.07 | -28.28 |
| Pakistan | 83647.03 | -155.32 | -0.19 | 81090.89 | 96.94 | 2711.47 | 3.24 |
| Palestine | 5732.57 | -16.55 | -0.29 | 4435.71 | 77.38 | 1313.41 | 22.91 |
| Philippines | 97995.09 | -90.31 | -0.09 | 131370.74 | 134.06 | -33285.34 | -33.97 |
| Qatar | 657.33 | -21.35 | -3.25 | 1360.73 | 207.01 | -682.05 | -103.76 |
| Republic of Korea | 274838.54 | 8888.59 | 3.23 | 310345.2 | 112.92 | -44395.25 | -16.15 |
| Saudi Arabia | 11800.44 | -458.17 | -3.88 | 14646.92 | 124.12 | -2388.3 | -20.24 |
| Singapore | 7807.89 | 296.84 | 3.8 | 33321.08 | 426.76 | -25810.03 | -330.56 |
| Sri Lanka | 52385.88 | -397.96 | -0.76 | 42343.87 | 80.83 | 10439.97 | 19.93 |
| Syrian Arab Republic | 17086.09 | 5.85 | 0.03 | 19314.53 | 113.04 | -2234.29 | -13.08 |
| Taiwan (Province of China) | 109030.8 | 4491.92 | 4.12 | 139339.53 | 127.8 | -34800.65 | -31.92 |
| Tajikistan | 1952.27 | 299.34 | 15.33 | 6308.76 | 323.15 | -4655.84 | -238.48 |
| Thailand | 253548.87 | 983.37 | 0.39 | 368758.14 | 145.44 | -116192.64 | -45.83 |
| Timor-Leste | 1552.31 | 78.75 | 5.07 | 1460.86 | 94.11 | 12.7 | 0.82 |
| Turkmenistan | 1262.16 | 119.91 | 9.5 | 4470.49 | 354.19 | -3328.25 | -263.69 |
| Türkiye | 350288.22 | -2871.68 | -0.82 | 388633.71 | 110.95 | -35473.81 | -10.13 |
| United Arab Emirates | 3966.5 | -506.52 | -12.77 | 14440.46 | 364.06 | -9967.44 | -251.29 |
| Uzbekistan | 9983.59 | 1183.54 | 11.85 | 31920.45 | 319.73 | -23120.41 | -231.58 |
| Viet Nam | 276568.35 | -1470.52 | -0.53 | 217119.04 | 78.5 | 60919.83 | 22.03 |
| Yemen | 14523.46 | 92.41 | 0.64 | 15537.73 | 106.98 | -1106.68 | -7.62 |
